# Podocalyxin‐Like Protein 1 Regulates Pluripotency through the Cholesterol Biosynthesis Pathway

**DOI:** 10.1002/advs.202205451

**Published:** 2022-11-14

**Authors:** Wei‐Ju Chen, Wei‐Kai Huang, Sarshan R. Pather, Wei‐Fang Chang, Li‐Ying Sung, Han‐Chung Wu, Mei‐Ying Liao, Chi‐Chiu Lee, Hsuan‐Hui Wu, Chung‐Yi Wu, Kuo‐Shiang Liao, Chun‐Yu Lin, Shang‐Chih Yang, Hsuan Lin, Pei‐Lun Lai, Chi‐Hou Ng, Chun‐Mei Hu, I‐Chih Chen, Chi‐Hsuan Chuang, Chien‐Ying Lai, Po‐Yu Lin, Yueh‐Chang Lee, Scott C. Schuyler, Axel Schambach, Frank Leigh Lu, Jean Lu

**Affiliations:** ^1^ Genomics Research Center Academia Sinica Genome and Systems Biology Degree Program College of Life Science National Taiwan University Taipei 10617 Taiwan; ^2^ Genomics Research Center Academia Sinica Taipei 11529 Taiwan; ^3^ Center for Genomic Medicine Massachusetts General Hospital Boston MA 02114 USA; ^4^ Cell and Molecular Biology Graduate Group Perelman School of Medicine University of Pennsylvania Philadelphia PA 19104 USA; ^5^ Institute of Biotechnology National Taiwan University Taipei 10617 Taiwan; ^6^ Agricultural Biotechnology Research Center Academia Sinica Taipei 11529 Taiwan; ^7^ Animal Resource Center National Taiwan University Taipei 10617 Taiwan; ^8^ Institute of Cellular and Organismic Biology Academia Sinica Taipei 11529 Taiwan; ^9^ Biomedical Translation Research Center (BioTReC) Academia Sinica Taipei 11529 Taiwan; ^10^ Department of Ophthalmology Hualien Tzu Chi Hospital Buddhist Tzu Chi Medical Foundation Hualien 97004 Taiwan; ^11^ Department of Biomedical Sciences College of Medicine Chang Gung University Division of Head and Neck Surgery Department of Otolaryngology Chang Gung Memorial Hospital Taoyuan 33302 Taiwan; ^12^ Institute of Experimental Hematology Hannover Medical School 30625 Hannover Germany; ^13^ Department of Pediatrics National Taiwan University Hospital and National Taiwan University Medical College Taipei 10051 Taiwan; ^14^ National RNAi Platform/ National Core Facility Program for Biotechnology Taipei 11529 Taiwan; ^15^ Department of Life Science Tzu Chi University Hualien 97004 Taiwan; ^16^ Graduate Institute of Medical Sciences National Defense Medical Center Taipei 11490 Taiwan

**Keywords:** cholesterol, extended pluripotency, integrin, podocalyxin‐like protein 1 (PODXL), primed stem cells

## Abstract

Deciphering signaling mechanisms critical for the extended pluripotent stem cell (EPSC) state and primed pluripotency is necessary for understanding embryonic development. Here, a membrane protein, podocalyxin‐like protein 1 (PODXL) as being essential for extended and primed pluripotency, is identified. Alteration of PODXL expression levels affects self‐renewal, protein expression of c‐MYC and telomerase, and induced pluripotent stem cell (iPSC) and EPSC colony formation. PODXL is the first membrane protein reported to regulate de novo cholesterol biosynthesis, and human pluripotent stem cells (hPSCs) are more sensitive to cholesterol depletion than fibroblasts. The addition of exogenous cholesterol fully restores PODXL knockdown‐mediated loss of pluripotency. PODXL affects lipid raft dynamics via the regulation of cholesterol. PODXL recruits the RAC1/CDC42/actin network to regulate SREBP1 and SREBP2 maturation and lipid raft dynamics. Single‐cell RNA sequencing reveals PODXL overexpression enhanced chimerism between human cells in mouse host embryos (hEPSCs 57%). Interestingly, in the human–mouse chimeras, laminin and collagen signaling‐related pathways are dominant in PODXL overexpressing cells. It is concluded that cholesterol regulation via PODXL signaling is critical for ESC/EPSC.

## Graphical abstract

Cholesterol is important for membrane composition and cell division. Chen et al. show that PODXL regulates de novo cholesterol biosynthesis and further sustains the primed and extended renewal states in hPSCs through c‐MYC, TERT, and ITGA2. PODXL overexpression facilitates the extended pluripotency state in vitro and in vivo. These data highlight that PODXL links cellular metabolism to stem cell self‐renewal.

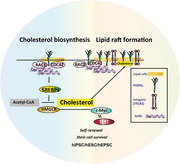

## 1 Introduction

Human embryonic stem cells (hESCs) are derived from the inner cell mass of the early embryo (blastocyst), which contains hundreds of cells. hESCs can further differentiate into all three germ layers (endoderm, mesoderm, and ectoderm) except trophectoderm (TE) which gives rise to placental cells after the embryo implantation.^[^ ^1^ ^]^ Conventionally cultured hESCs mimic post‐implantation epiblasts as compared to mouse ESCs and are usually considered to be in a “primed” state that cannot generate chimeras after injection into the inner cell mass (ICM) of embryos as that does in mESCs.^[^ ^2^ ^]^ Thus, the approaches to modify and optimize the essential components in the culture media can convert primed ESCs into naïve‐like pluripotent stem cells (PSCs), which are less differentiated and can form chimeras are warranted.^[^ ^3^ ^]^ For example, two research groups characterized the extended pluripotent stem cell (EPSC) state, which is a state similar to early‐stage embryos, in which cells can contribute to both the ICM and TE. The EPSCs form chimeras much more efficiently (~30%) compared to naïve stem cells (~0.5%) after injecting human cells into the mouse host embryo models.^[^ ^4^ ^]^ The discovery of these cells in the naïve state and in the EPSC state, which can contribute to chimeric mice, lays the foundation for the generation of interspecies organs from host embryos. For example, in the future, scientists may eventually use human EPSCs (hEPSCs) or naïve ESCs to generate human organs in pigs via interspecies blastocyst complementation.^[^ ^5^ ^]^ The patient somatic cells can at first be reprogrammed into induced pluripotent stem cells (iPSCs) by four factors (SOX2, OCT4, KLF4, c‐MYC). iPSCs share all the features of ESCs. iPSCs can be further converted to a naive state or extended PSCs, then injected into an animal embryo. The host embryo will be modified by knocking out a specific master regulator like PDX‐1, so the embryo fails to generate a pancreas.^[^ ^6^ ^]^ Then the iPSCs will form a pancreas in the host animals. Currently, hPSCs do not robustly contribute to chimera formation due to a lack of suitable culture conditions or the difference between human and animal embryos.^[^ ^7^ ^]^ To date, researchers have tried to understand the underlying mechanism of PSC self‐renewal and highly competent cells for survival and increased potency in host animals. Until now, very few ligand‐free transmembrane proteins have been implicated in sustaining pluripotency, except for epithelial cell adhesion molecule (EpCAM)^[^ ^8^ ^]^ and E‐cadherin in mouse ESCs,^[^ ^9^ ^]^ and Chromosome 9 Open Reading Frame 135 (C9ORF135) in hESCs.^[^ ^10^ ^]^ The functions of surface markers in maintaining self‐renewal and pluripotency of ESCs are mostly unknown. To this end, we generated hybridomas against the surface proteins of hPSCs to characterize surface proteins on hPSCs. We found many antibodies targeting Podocalyxin‐like Protein 1 (PODXL) which suggests that PODXL is the main surface antigen in hPSCs. PODXL can activate RAC1/CDC42 in cancers.^[^ ^11^ ^]^ RhoA family GTPases are important for PSC self‐renewal and survival. Dominant negative RAC1 counteracts Rho/ROCK signaling to induce apoptosis.^[^ ^12^ ^]^ RAC1/CDC42 can activate actin polymerization.

We found that PODXL can also activate the cholesterol pathway. Cholesterol is known to modulate the cell membrane physically.^[^ ^13^ ^]^ Cholesterol is also the main component of lipid rafts, serving as hubs of signal transduction, cytoskeletal organization, membrane trafficking, and pathogen entry in the cell membranes. Cholesterol biosynthesis is controlled by the SREBP transcription factor family via transcriptional regulation of critical rate‐limiting cholesterogenic proteins, e.g. 3‐Hydroxy‐3‐Methylglutaryl‐CoA Reductase (HMGCR). Sterol Regulatory Element‐Binding Protein 1c (SREBP1c) functions majorly in the liver and helps the fatty acid synthesis. Sterol Regulatory Element‐Binding Protein 1a (SREBP1a) drives both fatty acid synthesis and cholesterol synthesis in all tissues.^[^ ^14^ ^]^ In addition, one of the cholesterol master regulators is the related Sterol Regulatory Element‐Binding Protein 2 (SREBP2), which can activate the expression of HMGCR, HMGCS1, and mevalonate kinase (MVK).^[^ ^14^ ^]^

In this study, we found PODXL was required for hESC/iPSC/EPSC self‐renewal as well as iPSC and EPSC reprogramming. A membrane protein that directly controls cholesterol synthesis has not been reported. Here, We observed cholesterol is crucial for hESC renewal by demonstrating that the de novo cholesterol biosynthesis pathway was positively regulated by PODXL via the CDC42/RAC1/actin pathway for protein maturation of SREBP1 and SREBP2 to further sustain hESC/iPSC/EPSC self‐renewal. To explore the unknown role of PODXL‐dependent cholesterol regulation in maintaining pluripotency, we observed that the PODXL‐cholesterol pathway regulated c‐MYC and telomerase reverse transcriptase (TERT) expression and potentiated lipid raft formation. Furthermore, via relative quantitative proteomic analyses of lipid rafts, we found that PODXL also facilitated hPSC survival through the integrin *α*2 (ITGA2) pathway. Finally, we uncovered that inducing PODXL overexpression significantly increased human‐mouse interspecies chimerism in the peri‐implantation embryo stage (up to 57% hEPSCs). scRNA‐seq analyses also revealed the differentiation trajectory of PODXL‐overexpressing hEPSCs and the potential cell–cell and cell‐extracellular matrix (ECM) contacts that are critical for interspecies chimerism.

## 2 Results

### 2.1 Generation and Characterization of Monoclonal Antibodies That Recognize Cell Surface Antigens on hPSCs

**Figure 1:**
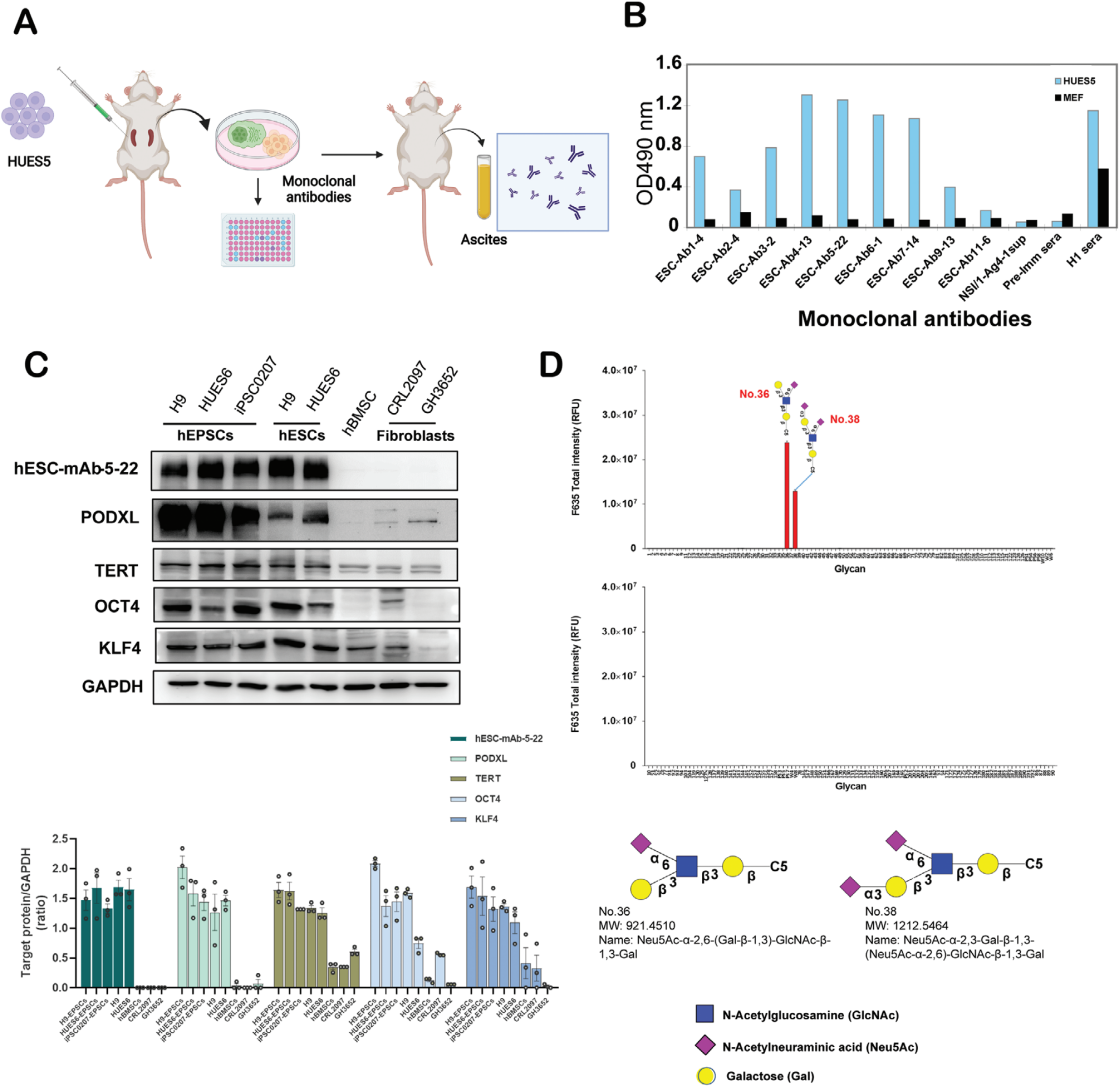
Generation and characterization of monoclonal antibodies (mAbs) against hESCs. A) Schematic of the monoclonal antibody screening process and generation of antibodies against hESCs. (Created with BioRender.com.) B) ELISA analyses of the binding abilities of the mAbs ESC‐Ab‐1‐4, ‐2‐4, ‐3‐2, ‐4‐13, ‐5‐22, ‐6‐1, ‐7‐14, ‐9‐13, and ‐11‐6 against HUES5 cells. Hybridoma supernatants were added onto HUES5‐coated wells. Sera from BALB/c mice were challenged with HUES5 and were used as a positive control. NSI/1‐Ag4‐1 culture media served as a negative control. C) Results of the quantification of Western blots show the expression levels of targeted proteins in a variety of cell lines. hEPSCs, human extended pluripotent stem cells. hESCs, human embryonic stem cells. hBMSCs, human bone marrow stem cells. Error bars indicate the averages ± SDs (*n* = 3), D) Results of glycan array analyses showing the glycans bound by ESC‐Ab‐5‐22 at 10 µg mL^−1^. GalNAc: N‐Acetylgalactosamine, GlcNAc: N‐Acetylglucosamine, Neu5Ac: N‐Acetylneuraminic acid, Gal: Galactose.

To identify membrane proteins on hESCs, we used hESCs as the antigen to generate hybridomas. After screening 20 hybridoma clones (**Figure** **1**A), nine clones exhibited high binding affinity to HUES5 cells in cellular ELISA assays (Figure 1B). The isotypes of selected monoclonal antibodies (mAbs) against HUES5 cells were IgM (4/9), IgG3 (3/9), and IgG1 (2/9) (Figure S1A, Supporting Information). Flow cytometry showed strong mAb binding to undifferentiated HUES5 cells, but not its differentiated counterparts, especially for hESC‐mAb‐5‐22 (Figure S1B, Supporting Information). mAb‐hESC‐Ab‐5‐22 also recognized cells in the hEPSC state grown in LCDM media (LCDM‐EPSCs) and primed hESCs but not human bone marrow stem cells (hBMSCs), human neonatal foreskin fibroblast (CRL‐2097) or adult dermal fibroblast (GH‐3852) as determined by Western blotting (Figure 1C and Figure S1C, Supporting Information), providing evidence for specific binding by the mAb‐hESC‐Ab‐5‐22 antibody to undifferentiated hPSCs. We also isolated the mAb‐hESC‐Ab‐1‐4, mAb‐hESC‐Ab‐3‐2, mAb‐hESC‐Ab‐4‐13, mAb‐hESC‐Ab‐6‐1, and mAb‐hESC‐Ab‐11‐6 clones which also recognized the mAb‐hESC‐Ab‐5‐22 co‐immunoprecipitated proteins of the same molecular weights, suggesting these monoclonal antibodies recognize the same or similar proteins that are enriched in hESCs (Figure S1D, Supporting Information). The target protein of mAb‐hESC‐Ab‐5‐22 was immunoprecipitated from HUES5 lysates and subjected to LC‐MS/MS analyses. One protein containing multiple peptides identified by LC‐MS/MS was PODXL (peptides in red highlights, Figure S1E, Supporting Information). Thus, PODXL is likely the main surface antigen of hESCs. Since PODXL is a highly sialylated type I membrane protein, we wanted to know whether mAb‐hESC‐Ab‐5‐22 could also recognize glycans on PODXL. Neu5Ac‐*α*‐2,6‐(Gal‐*β*‐1,3)‐GlcNAc‐*β*‐1,3‐Gal and Neu5Ac‐*α*‐2,3‐Gal‐*β*‐1,3‐(Neu5Ac‐*α*‐2,6)‐GlcNAc‐*β*‐1,3‐Gal were identified as two major glycans recognized by mAb‐hESC‐Ab‐5‐22 (Figure 1D and Figure S1F, Supporting Information). We next investigated PODXL's expression profile in human early embryos and found PODXL was abundantly expressed from the 1‐cell to the 4‐cell stages and moderately expressed from the 8‐cell to the blastocyst stages (Figure S1G, Supporting Information). The expression profile of *PODXL* differed from that of other pluripotent markers, e.g., *OCT4*, *LIN28A*, *SOX2*, *NANOG*, and *KLF4*, which were highly expressed only after the 8‐cell stage (Figure S1G, Supporting Information), consistent with previous observations.^[^ ^15^ ^]^

### 2.2 PODXL Is Essential for Primed hPSC Self‐Renewal, iPSC Formation, and EPSC Self‐Renewal

**Figure 2:**
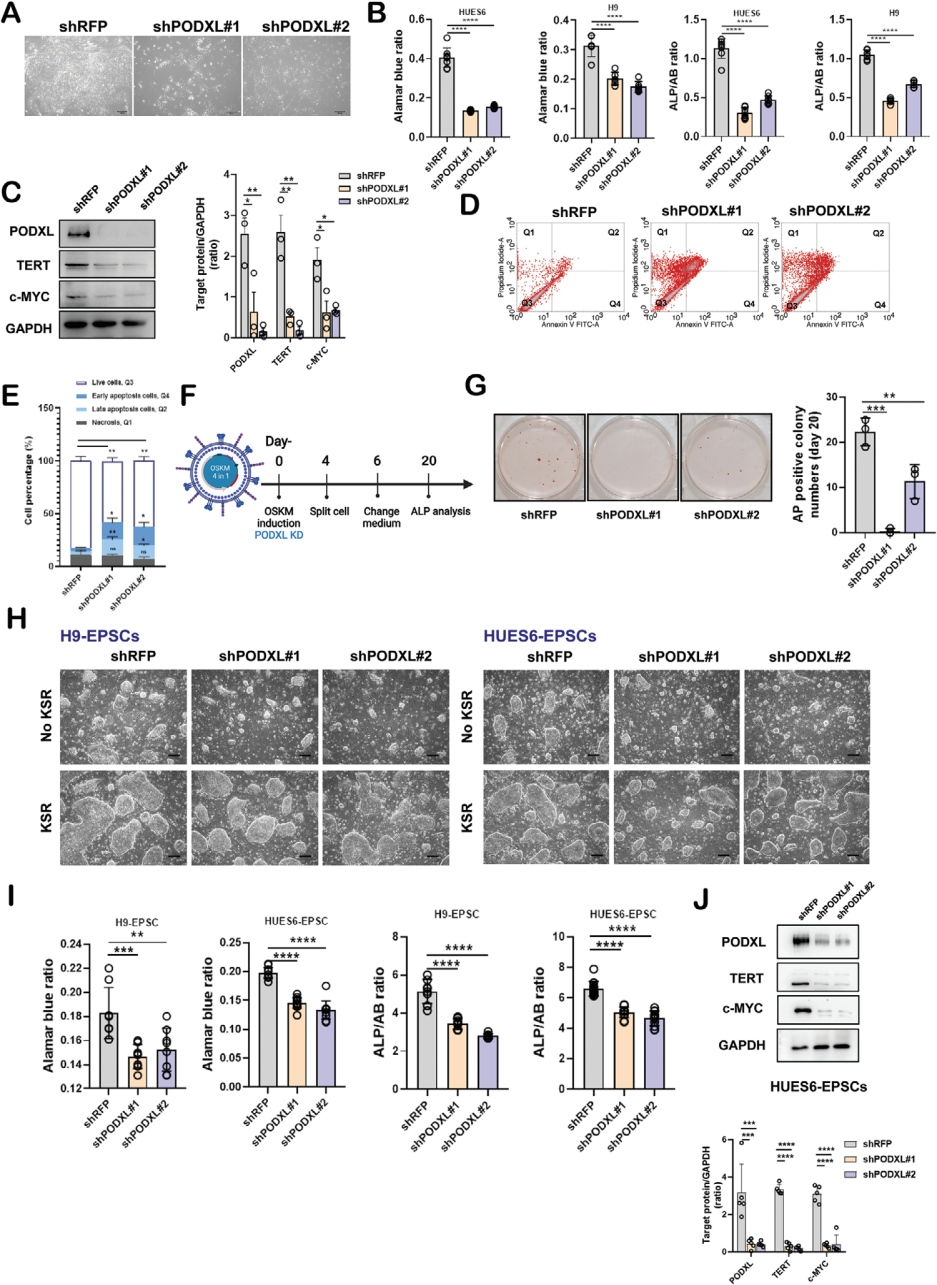
PODXL is necessary for hPSC self‐renewal and viability. A) Bright‐field images of shRNA‐treated HUES6 hESCs. Scale bar, 200 µm. B) Alamar blue (AB) assays and ALP activity assays (a pluripotent marker) were performed in shRNA‐treated HUES6 and H9 cells. ALP levels were normalized to the relative cell numbers measured in the AB assays. The error bars indicate the averages ± SDs (*n* = 8 in HUES6; *n* = 6 in H9). P‐values were determined with one‐way ANOVA (****p < 0.0001) with Dunnett's multiple comparison test performed relative to shRFP hESCs. C) Quantification of c‐MYC and TERT in shRNA‐treated HUES6 measured by Western blotting. The error bars indicate the averages ± SEMs (*n* = 3). P‐values were determined with one‐way ANOVA (**p < 0.01, *p < 0.05) with Dunnett's multiple comparison test performed relative to shRFP hESCs. D) Measurements of apoptosis/necrosis, as demonstrated by fluorescence‐assisted cell sorting (FACS) analyses using annexin V‐PI in HUES6. E) Quantification of annexin V‐ and PI‐positive cells. The error bars indicate the averages ± SEMs (*n* = 4). P‐values were determined with one‐way ANOVA (**p < 0.01, *p < 0.05) with Dunnett's multiple comparison test performed relative to shRFP hESCs. F) Flow chart of iPSC generation. (Created with BioRender.com) CRL‐2097 fibroblasts were stably infected with lentivirus expressing shRNAs and lentivirus encoding four reprogramming factors at the same time. 4 days after infection, cells were split into matrigel‐coated plates in a feeder‐free condition. G) Reprogrammed colonies were stained to assess ALP, and ALP‐positive colonies were counted. After 20 days of virus induction, ALP staining was performed and ALP‐positive colonies were counted with Image J software. The error bars indicate the averages ± SDs (*n* = 3). P‐values were determined with one‐way ANOVA (***p < 0.001, **p < 0.01) with Dunnett's multiple comparison test performed relative to shRFP hESCs. H) Bright‐field images of shRNA‐treated H9 and HUES6‐derived EPSCs. KSR: 5% KnockOut serum replacement. I) AB assays and ALP activities were assessed in shRNA‐treated HUES6‐ or H9‐derived hEPSCs. The ALP activity levels were normalized to the relative cell numbers (AB assays). The error bars indicate the averages ± SDs (*n* = 8 in H9‐EPSCs; *n* = 12 in HUES6‐EPSCs). P‐values were determined with one‐way ANOVA (****p < 0.0001, ***p < 0.001, **p < 0.01) with Dunnett's multiple comparison test performed relative to shRFP hESCs. J) c‐MYC and TERT were downregulated after PODXL knockdown in HUES6‐derived EPSCs as observed by Western blotting. The error bars indicate the averages ± SEMs (*n* = 5). P‐values were determined with one‐way ANOVA (****p < 0.0001, ***p < 0.001) with Dunnett's multiple comparison test performed relative to shRFP hESCs.

To assess the function of PODXL in hPSCs, we used two different shRNAs to knock down PODXL in HUES6 cells. After shRNA knockdown, cell colonies were barely maintainable and displayed differentiated phenotypes (**Figure** **2**A), with all decreasing the relative cell number (as determined by Alamar blue assays), the levels of the pluripotency marker alkaline phosphatase (ALP), and the protein expression levels of c‐MYC and TERT (Figure 2B,C). Cell apoptosis was increased in shPODXL HUES6 hESCs compared to shRFP HUES6 hESCs as measured by Annexin V‐propidium iodide (PI) analyses (Figure 2D,E). These data suggest that PODXL knockdown triggered cell death and inhibited the renewal of hPSCs. By contrast, the relative cell numbers, ALP activity, and c‐MYC and TERT protein levels were all increased upon PODXL overexpression (Figure S2A–C, Supporting Information).

To study the functional roles of PODXL in the onset of iPSC reprogramming, human foreskin fibroblasts were co‐treated with shPODXL or PODXL overexpression viruses and lentiviral vectors encoding four transcription factors (OCT3/4, KLF4, SOX2, and c‐MYC, called “OKSM”) (Figure 2F and Figure S2D, Supporting Information). Cells treated with shPODXL had a much lower colony formation efficiency compared to shRFP‐treated cells (Figure 2G), while cells transducted with PODXL‐overexpressing viruses displayed an increase in the iPSC colony numbers compared to cells treated with GFP‐overexpressing viruses (Figure S2E, Supporting Information), suggesting that PODXL was required for the acquisition of pluripotency by somatic cells. We also confirmed that loss‐of‐function phenotypes, such as a decrease in the relative cell numbers, ALP activity, and c‐MYC and TERT protein expression, were fully rescued by transgenic PODXL re‐expression in PODXL knockdown cells (Figure S2F–H, Supporting Information). Thus, the phenotypic changes in shPODXL‐expressing cell lines were caused by the loss of PODXL expression and not by off‐target effects.

In molecular signatures and cultural conditions, hESCs mimic mouse primed stem cells from epiblasts at the post‐implantation stage of embryogenesis. Recently, Yang et al. discovered that the molecular characteristics of the human EPSC state are more similar to the early stages of human pre‐implantation embryos and with a higher chimera rate compared to the primed state hESCs.^[^ ^4a^ ^]^ We are interested in how PODXL functions in hEPSCs. After PODXL knockdown under a serum‐free condition, we found that the relative cell numbers and ALP activity, as well as c‐MYC and TERT protein expression levels, were decreased in EPSCs (Figure 2H–J). We also observed that HUES6 and H9 cells in the hEPSCs state cultured in an N2B27‐LCDM medium containing 5% KnockOut serum replacement (KSR) did not exhibit the inhibitory effects of shPODXL (Figure 2H). To further assess the function of PODXL in extended pluripotency, we first overexpressed PODXL in H9 hESCs and then transferred the H9 hESCs to N2B27‐LCDM media. The numbers of typical dome‐shaped colonies were increased in comparison with those in the GFP control group on day 8 of the EPSC state reprogramming (Figure S2I, Supporting Information). The colony sizes and cell expansion numbers were increased after PODXL overexpression with or without KSR in HUES6 and H9 derived EPSCs (Figure S2J,K, Supporting Information), and the c‐MYC and TERT protein expression levels were increased, suggesting that PODXL also promoted the EPSC state of self‐renewal (Figure S2L, Supporting Information). Taken together, our data demonstrate that PODXL plays an important role in the maintenance of primed pluripotency, the induction of iPSCs, and the acquisition of extended pluripotency.

### 2.3 PODXL Regulates Cellular Cholesterol Required for hPSC Pluripotency and the Addition of Exogenous Cholesterol Rescues shPODXL Knockdown Phenotypes

**Figure 3:**
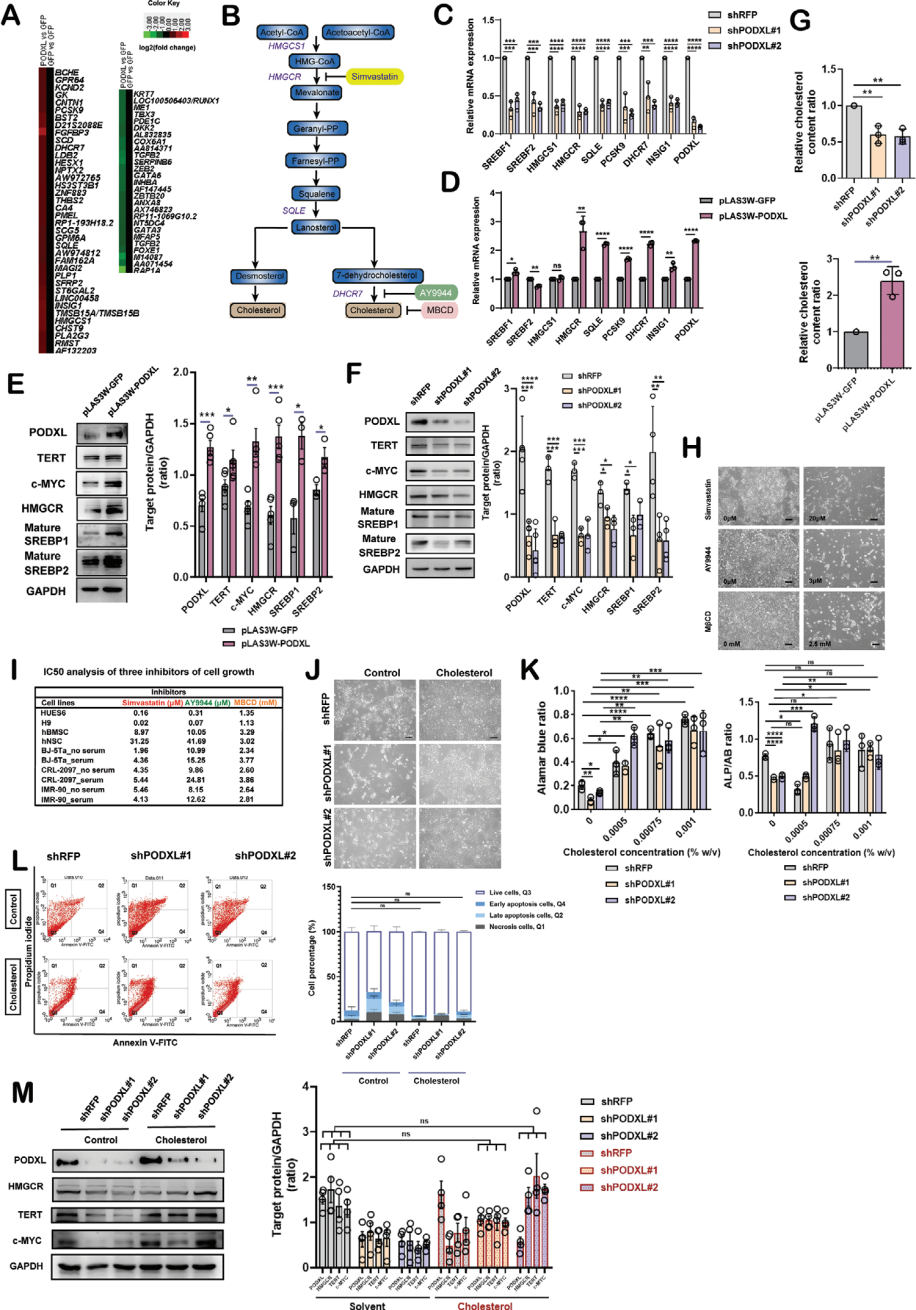
PODXL regulates de novo cholesterol biosynthesis and exogenous addition of cholesterol rescues PODXL knockdown‐dependent loss of pluripotency. A) Gene expression analyses of PODXL‐overexpressing HUES6 hESCs using cDNA microarrays. B) Schematic of the cholesterol biosynthesis pathway. Inhibitors used in this study are illustrated targeting each specified enzyme. C,D) mRNA levels of cholesterol genes were analyzed by qRT‐PCR in shPODXL HUES6 hESCs. The error bars indicate the averages ± SEMs (*n* = 3). P‐values were determined with one‐way ANOVA (****p < 0.0001, ***p < 0.001, **p < 0.01, *p < 0.05) with Dunnett's multiple comparison test performed relative to shRFP hESCs. For PODXL‐overexpressing hESCs, the error bars indicate the averages ± SEMs (*n* = 3), and *p*‐values were determined with an unpaired Student's t‐test (****p < 0.0001, ***p < 0.001, **p < 0.01, *p < 0.05). E) Quantification of Western blots showing HMGCR, SREBP1, SREBP2, c‐MYC, and TERT levels in PODXL‐overexpressing HUES6 cells. The error bars indicate the averages ± SEMs (PODXL, TERT, c‐MYC, HMGCR, and GAPDH, *n* = 5; SREBP1 and SREBP2, *n* = 3). P‐values were determined with an unpaired Student's t‐test (***p < 0.001, **p < 0.01, *p < 0.05). F) Quantification of Western blots showing HMGCR, SREBP1, SREBP2, c‐MYC, and TERT levels in shPODXL‐transduced HUES6 cells. The error bars indicate the averages ± SEMs (*n* = 3). P‐values were determined with one‐way ANOVA (****p < 0.0001, ***p < 0.001, **p < 0.01, *p < 0.05) with Dunnett's multiple comparison test performed relative to shRFP hESCs. G) Cellular cholesterol content was determined with an Amplex Red assay kit. The error bars indicate the averages ± SEMs (*n* = 3). P‐values were determined with one‐way ANOVA (**p < 0.01) with Dunnett's multiple comparison test performed relative to shRFP hESCs. For PODXL overexpression, *p*‐values were determined with an unpaired Student's t‐test (**p < 0.01) relative to GFP control cells. H) Bright‐field images of HUES6 hESCs treated separately with simvastatin, AY9944, and MBCD for 3 days. Scale bar, 200 µm. I) IC_50_ analyses of the three inhibitors in hESCs, hBMSCs, hNSCs, and fibroblasts assessed by cell growth (AB assays) under serum‐free or serum‐supplemented conditions. J) Bright‐field images of HUES6 hESCs with PODXL knockdown supplemented with cholesterol for 7 days. Scale bar, 200 µm. K) Alamar blue and ALP activity assays were performed in HUES6 hESCs with PODXL knocked down supplemented with cholesterol. The error bars indicate the averages ± SDs (*n* = 3). P‐values were determined with one‐way ANOVA with Dunnett's multiple comparison test (****p < 0.0001, ***p < 0.001, **p < 0.01, *p < 0.05) performed relative to shRFP hESCs in solvent condition (0). L) Quantification of Annexin V‐/PI‐positive cells in HUES6 hESCs with PODXL knocked down supplemented with cholesterol. The error bars indicate the averages ± SEMs (*n* = 3). P‐values were determined with one‐way ANOVA with Dunnett's multiple comparison test (ns, not significant) performed relative to shRFP hESCs in solvent condition (0% w/v). M) Quantification of Western blots was performed in HUES6 hESCs with PODXL knocked down supplemented with cholesterol for 7 days. The error bars indicate the averages ± SEMs (*n* = 4). P‐values were determined with one‐way ANOVA (ns, not significant) with Dunnett's multiple comparison test performed relative to shRFP hESCs in solvent condition (0% w/v) for each of the target proteins.

To map the early signals triggered by PODXL, comprehensive gene expression profile changes after 3 days of PODXL overexpression were analyzed by cDNA microarrays. Via the application of the Database for Annotation, Visualization and Integrated Discovery (DAVID) functional annotation clustering tools,^[^ ^16^ ^]^ we found that the upregulated gene sets triggered by PODXL overexpression were significantly enriched in cholesterol‐related biosynthesis pathways and membrane‐associated clusters, whereas the downregulated gene clusters were enriched in pathways involved in the regulation of RNA metabolic processes, morphogenesis and transcription factor activity (Figure S3A, Supporting Information). In the genes whose levels changed more than two‐fold, 38 genes were upregulated and 26 were downregulated (**Figure** **3**A). Among the upregulated genes were the cholesterol‐related genes 3‐Hydroxy‐3‐Methylglutaryl‐CoA Synthase 1 (*HMGCS1*), 7‐Dehydrocholesterol Reductase (*DHCR7*), Squalene Epoxidase (*SQLE*), Protein Convertase Subtilisin/Kexin Type 9 (*PCSK9*), Insulin‐Induced Gene 1 (*INSIG1*), and 3‐Hydroxy‐3‐Methylglutaryl‐CoA Reductase (*HMGCR*) (with changes of up to 1.6‐fold) involved in choletsterol biosynthesis (Figure 3A). In addition, the downregulated gene set included the differentiation‐related genes *TGFB2*, *ZEB2*, *GATA6*, *GATA3*, and *FOXE1* (Figure 3A). The transcriptomic changes in the cholesterol biosynthesis pathway were also confirmed in shPODXL knockdown HUES6 hESCs by bulk RNA‐seq (Figure S3B, Supporting Information). qRT‐PCR was done to verify the cholesterol gene expression patterns in shPODXL (Figure 3C) and PODXL overexpressing cells (Figure 3D). To explore the potential mechanism(s) by which PODXL affected cellular cholesterol homeostasis, we focused on the rate‐limiting enzyme in cholesterol synthesis HMGCR. We found HMGCR mRNA and HMGCR protein levels were proportionally altered upon PODXL knockdown and overexpression (Figure 3C–F). Accordingly, SREBP1 and SREBP2 were decreased after PODXL knockdown in hPSCs and EPSCs, while the levels of both SREBP1 and SREBP2 were increased upon PODXL overexpression in both primed hESCs and EPSCs (Figure 3E–F, Figures S2L and S3C, Supporting Information).

To understand how PODXL affected cholesterol metabolism genes, the total cellular cholesterol content was measured decreased in PODXL knockdown cells and upregulated in cells with PODXL overexpression (Figure 3G and Figure S3D, Supporting Information). In addition, HMGCR knockdown cells produced flattened and less compact colonies phenotypically similar to those of shPODXL‐expressing cells (Figure S3E, Supporting Information). Consistent with this finding, reduced relative cell numbers and ALP activity were observed in shHMGCR‐treated hESCs, and c‐MYC and TERT protein levels were decreased after HMGCR knockdown (Figure S3F,G, Supporting Information). These data suggest that PODXL can regulate cholesterol homeostasis through the cholesterol metabolism gene, HMGCR. To examine the effects of cholesterol on pluripotency, simvastatin and AY9944 were used to block cholesterol biosynthesis, and methyl‐*β*‐cyclodextrin (MBCD) was used to deplete cellular cholesterol (Figure 3H).^[^ ^17^ ^]^ We observed that the cell morphology changed quickly (within ~24 h) and ALP activity was decreased upon treatment with these inhibitors (Figure 3H and Figure S3H, Supporting Information). Inhibition of cholesterol synthesis by simvastatin downregulated TERT, c‐MYC, HMGCR, and PODXL expression (Figure S3I, Supporting Information). We sought to determine whether stem cells were more reliant on the cholesterol pathway than adult stem cells, such as human neural stem cells (hNSCs), human bone marrow stem cells (hBMSCs), and somatic cells. Toward this end, the IC_50_ of each inhibitor in each cell type was measured compared to hESCs. We observed that hPSCs were more sensitive to cholesterol inhibition compared to adult stem cells and somatic fibroblasts (by 3.22‐ to 1562.5‐fold) (Figure 3I).

We then examined if PODXL regulated pluripotency mainly through cholesterol. We first overexpressed PODXL for one day and then separately treated cells with simvastatin, AY9944, and MBCD. PODXL‐overexpressing cells showed enhanced cell growth and ALP activity, and cells separately pulsed with the three inhibitors showed dose‐dependent reductions in the relative cell numbers and ALP activity levels compared with those of the solvent‐treated only control in HUES6 cells (Figure S3J, Supporting Information). Free cholesterol levels were also decreased in PODXL‐overexpressing cells after treatment with simvastatin, AY9944, and MBCD (Figure S3K, Supporting Information). These results indicate that inhibition of cholesterol synthesis blocks PODXL‐induced self‐renewal and that cholesterol is the downstream target of PODXL. Exogenous cholesterol supplementation prevented morphological changes resulting from PODXL knockdown (Figure 3J). Notably, the reduced relative cell numbers, ALP activity levels, increased cell apoptosis as well as downregulation of HMGCR, c‐MYC and TERT protein expression levels induced by PODXL knockdown were all restored by cholesterol replenishment (Figure 3K,L). These results suggest that PODXL regulates pluripotency mainly through regulating cholesterol metabolism. Cholesterol supplementation could also enhance iPSC reprogramming efficiency (Figure S3L, Supporting Information), underpinning the importance of cholesterol for hPSC self‐renewal.

### 2.4 PODXL Recruits the RAC1/CDC42/Actin Network to Regulate SREBP1 and SREBP2 Maturation and Lipid Raft Dynamics

**Figure 4:**
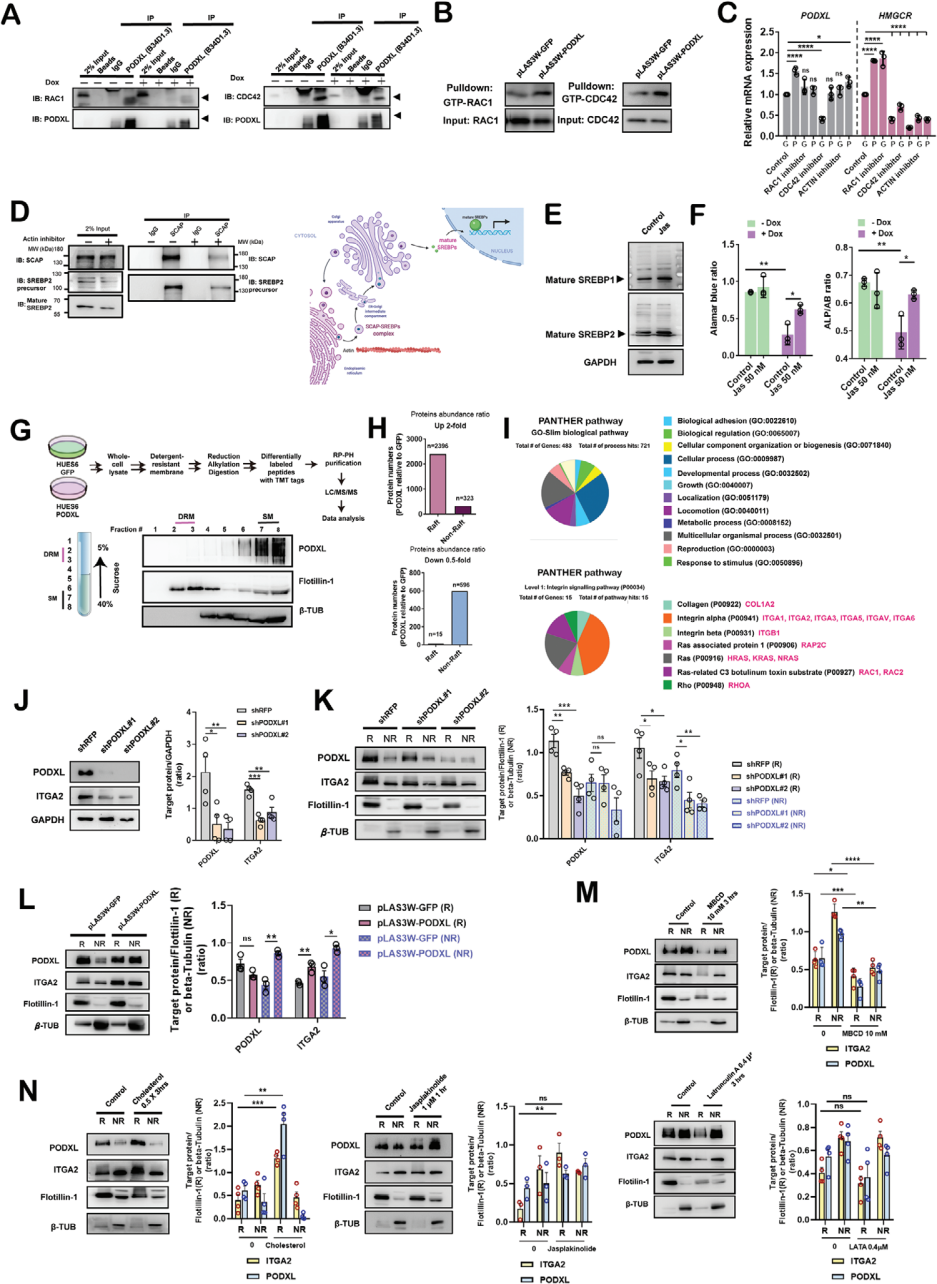
PODXL recruits the RAC1/CDC42/actin network to regulate SREBP1 and SREBP2 maturation and lipid raft dynamics. A) Western blots showing co‐immunoprecipitations between PODXL and endogenous protein of RAC1 and CDC42 in hiPSCs. The inducible *PODXL* knockout was induced after doxycycline (+Dox, 2 × 10^−6^ m) treatment. B) Upregulation of RAC1/CDC42 activity was detected by pull‐down assays using GST‐PBD fusion protein magnetic beads after forced PODXL expression in HUES6 hESCs. C) qRT‐PCR analyses of *HMGCR* mRNA expression after RAC1, CDC42, and actin inhibition in HUES6 cells. The error bars indicate the averages ± SDs (*n* = 3). P‐values were determined with one‐way ANOVA (***p < 0.001, **p < 0.05, *p < 0.05) with Dunnett's multiple comparison test performed relative to GFP control hESCs in solvent conditions. D) Western blots showing co‐immunoprecipitations between SCAP and endogenous protein of SREBP2 in hPSCs. HUES6 cells were treated with or without Latrunculin A (actin inhibitor). (Created with BioRender.com), E) Western blots showing upregulation of SREBP1 and SREBP2 after Jasplakinolide treatment (1 × 10^−6^ m, 1 hour) in HUES6 hESCs. F) Activation of actin polymerization by Jasplakinolide (Jas) can rescue the downregulation of relative cell numbers and ALP activity in Dox‐inducible *PODXL* knockout hiPSCs. The error bars indicate the averages ± SDs (*n* = 3). P‐values were determined with an unpaired Student's t‐test (**p < 0.01, *p < 0.05) relative to control cells. G) Schematic of lipid raft preparation. Fractions 2–3, lipid rafts (DRM); fractions 7–8, non‐raft (SM) fraction. Flotillin‐1, lipid raft marker; *β*‐TUBULIN, non‐raft marker. 30 µL of each fraction (0.5 mL/fraction) was loaded. H) Protein abundance ratios with changes of greater than 2‐fold and less than 0.5‐fold are shown in TMT‐labeled mass spectrometry analyses. I) Proteins with a change in the abundance ratio in lipid rafts greater than 4‐fold subjected to PANTHER pathway analyses. J) Quantification of Western blots showing ITGA2 in shPODXL‐treated HUES6 hESCs. Error bars indicate the averages ± SEMs (*n* = 4). P‐values were determined with one‐way ANOVA (***p < 0.001, **p < 0.01, *p < 0.05) with Dunnett's multiple comparison test. K) Quantification of the ITGA2 protein levels in lipid rafts and non‐raft fractions in PODXL knockdown HUES6 hESCs. Protein expression in the raft fraction (R) was normalized to Flotillin‐1 expression; in the non‐raft fraction (NR), protein levels were normalized to *β*‐TUBULIN (*β*‐TUB). The error bars indicate the averages ± SEMs (*n* = 4). P‐values were determined with one‐way ANOVA (***p < 0.001, **p < 0.01, *p < 0.05) with Dunnett's multiple comparison test. L) Quantification of the ITGA2 protein levels in lipid raft and non‐raft fractions in PODXL‐overexpressing hESCs. The error bars indicate the averages ± SEMs (*n* = 3). P‐values were determined with an unpaired Student's t‐test (**p < 0.01, *p < 0.05) relative to the corresponding control group. M) Western blots showing the dysregulation of lipid rafts and ITGA2 after cholesterol deprivation (MBCD, 10 m m, 3 h) or actin depolymerization (Latrunculin A, 0.4 × 10^−6^ m, 3 h) in HUES6 hESCs. The error bars indicate the averages ± SEMs (*n* = 4 in MBCD; *n* = 4 in Latrunculin A). P‐values were determined with an unpaired Student's t‐test (**p < 0.01, *p < 0.05) relative to the corresponding solvent control group (0). N) Western blots show that cholesterol supplementation (0.5x of cholesterol concentrate [0.0005% w/v], 3 h) or induced actin polymerization (Jasplakinolide) can increase ITGA2 expression in HUES6 hESCs. The error bars indicate the averages ± SEMs (*n* = 4 in cholesterol; *n* = 3 in Jasplakinolide). P‐values were determined with an unpaired Student's t‐test (***p < 0.01, **p < 0.01, *p < 0.05) relative to the corresponding solvent control group (0).

PODXL was found to directly bind with cortactin and its activity was induced by RAC1/CDC42 activation to regulate the actin cytoskeleton in cancer cells.^[^ ^11^ ^]^ We designed an inducible CRISPR/Cas9 genome editing method in a doxycycline (Dox) responsive Cas9 expression system to knockout PODXL in human iPSCs (Figure S4A, Supporting Information).^[^ ^18^ ^]^ Human iPSCs had smaller colonies, lower relative cell numbers, and lower ALP activity levels after induction of wild‐type Cas9 expression (CRISPRn) upon doxycycline treatment for 3 and 5 days compared to the solvent‐only control cells (Figure S4B–D, Supporting Information). The levels of the c‐MYC and TERT proteins were also decreased after inducible PODXL knockout after 4 days in Essential 8 (E8) media (Figure S4E, Supporting Information). The deletion of a genomic region containing the first exon of PODXL was confirmed by PCR (Figure S4F, Supporting Information). In co‐immunoprecipitation assays, RAC1 and CDC42 could associate with PODXL, but these interactions declined under Dox‐induced PODXL knockout conditions (**Figure** **4**A). RAC1 and CDC42 activation were both upregulated in PODXL overexpressing HUES6 hESCs as measured by a GTP‐bound pull‐down assay (Figure 4B). These data indicate that PODXL can associate and activate RAC1 and CDC42 activities in hPSCs. RAC1 and CDC42 have been known to promote actin polymerization, and we found that the mRNA level of HMGCR was downregulated after treatment with an actin inhibitor (Figure 4C). We also determined that PODXL overexpression can enhance actin polymerization and inhibition of RAC1 and CDC42 can reverse it (Figure S4G–I, Supporting Information). For example, the colocalization of PODXL and RAC1, PODXL and F‐actin, and RAC1 and F‐actin were all enhanced in PODXL‐overexpressing hESCs (Figure S4J, Supporting Information). These data support the hypothesis that PODXL links RAC1 and CDC42 to regulate actin polymerization.

Cholesterol regulation is mainly mediated by the SREBP cleavage‐activating protein (SCAP)/SREBP2 complex. SCAP stabilizes SREBPs and escorts SREBPs to help transport them to the Golgi for further maturation processing. In the Golgi, two steps of proteolytic cleavage release the N‐terminal domain of SREBPs and which then travel to the nucleus and activate lipogenic gene transcription. We set out to test if PODXL regulated the cleavage of SREBPs into the mature forms by modulating actin polymerization. Co‐Immunoprecipitation of SCAP protein showed that both SCAP protein, the SREBP2 precursor, and mature SREBP2 were downregulated after inhibiting actin polymerization (Figure 4D). These data indicate that actin polymerization was required for SCAP‐mediated SREBP stabilization and transportation. On the other hand, actin polymerization promoted by Jasplakinolide induced upregulation of mature forms of SREBP1 and SREBP2 (Figure 4E). Activation of actin polymerization could also partially restore the loss of self‐renewal upon induced PODXL knockout in the attached cells (Figure 4F). Collectively, these results reveal that PODXL regulates the maturation of SREBPs through activating the RAC1/CDC42/actin cytoskeletal network in hESCs.

Cholesterol plays a physical role in the maintenance of membrane fluidity and permeability and serves as a building block for lipid raft formation. It has been shown that cholesterol concentrations are proportional to the abundance of lipid rafts in the cell membrane fraction.^[^ ^19^ ^]^ The abundance of lipid rafts in the membrane fraction was decreased after PODXL knockdown; in contrast, increased levels of lipid rafts were observed after PODXL overexpression (Figure S4K, Supporting Information). In addition, to check if the PODXL could localize in lipid rafts, confocal overlay image analysis was performed. In PODXL overexpressing cells, patch‐like PODXL (pink dots) signals and GM1 (lipid raft marker, green dots) overlapping signals were increased (Figure S4L, Supporting Information). These results suggest that PODXL plays a positive regulatory role in lipid raft formation.

To gain a more comprehensive view of PODXL regulation of lipid rafts, we conducted tandem mass tag (TMT) proteomic characterization of protein profile changes in lipid raft‐enriched and non‐raft membranes from PODXL‐overexpressing HUES6 hESCs (Figure 4G). The detergent‐resistant membrane (DRM) and detergent‐soluble membrane (SM) fractions were isolated by a sucrose gradient ultracentrifugation method.^[^ ^20^ ^]^ High confident peptide identifications were chosen (4433 proteins), among which 2396 were enriched in lipid raft fractions and 323 were enriched in non‐raft fractions (Figure 4H). In contrast, 15 of the lipid raft‐associated proteins and 596 of the non‐raft proteins were decreased in tested fractions (Figure 4H). These data suggest that PODXL overexpression essentially increases the expression of lipid raft‐associated proteins and concurrently decreases the expression of non‐raft proteins.

To identify the signaling pathways in which PODXL participates through lipid rafts, 483 proteins (fold change > 4) were subjected to PANTHER gene ontology (GO) enrichment analyses. The biological function‐related terms “cellular process,” “locomotion,” and “multicellular organismal process” were enriched (Figure 4I). Among lipid raft‐associated proteins that increased in the PODXL‐raft fraction, 15 proteins (3.1%) are involved in the integrin signaling pathway (Figure 4I). These data indicate that PODXL recruits cytoplasmic RAC proteins to execute signaling accompanied by integrin pathways in lipid rafts. For instance, ITGA2 protein levels decreased in hESCs after PODXL knockdown (Figure 4J). And the expression levels of ITGA2 in lipid raft and non‐raft fractions were decreased after PODXL knockdown and enriched after PODXL overexpression (Figure 4K,L).

We also performed cholesterol depletion via treatment with MBCD and inhibition of actin polymerization by Latrunculin A to investigate the ITGA2 dynamics between the lipid raft and non‐raft fractions in hESCs. Both cholesterol depletion and inhibition of actin polymerization decreased the expression of the lipid raft marker flotillin‐1, showing the disruption of lipid rafts (Figure 4M). Cholesterol depletion also reduced ITGA2 in rafts (Figure 4M). Actin inhibition decreased ITGA2 in rafts at a border line level (P = 0.06). However, cholesterol supplementation and activation of actin polymerization by Jasplakinolide yielded increases in ITGA2 in rafts (Figure 4N). These data imply cholesterol and actin were required for ITGA2 enrichment in lipid rafts and indicate that the actin cytoskeleton and cholesterol are critical for lipid raft dynamic formation in hESCs. PODXL orchestrates actin cytoskeleton activity and cholesterol biosynthesis to initiate lipid raft formation as measured by an increase in the lipid raft protein ITGA2. These observations are supported by previous studies demonstrating that cytoskeletal activity regulated the spatiotemporal lateral distribution of lipids in membranes and that lipid distribution, in turn, determined the protein distribution at the membrane surface.^[^ ^21^ ^]^

### 2.5 Inducing PODXL Overexpression Promotes the Extended Pluripotency State of hEPSC Cells

**Figure 5:**
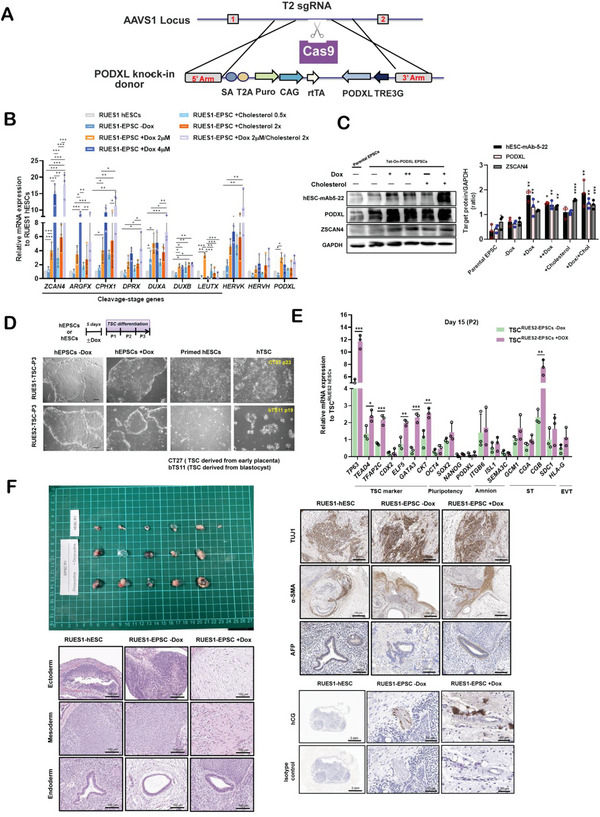
Inducing PODXL overexpression extends pluripotency potential in vitro and teratoma formation in vivo. A) Schematic view of the setup of an inducible Tet‐on‐PODXL vector in hEPSCs. B) qRT‐PCR assays accessing the cleavage‐stage specific gene expression levels in RUES1 derived‐hEPSCs after inducing PODXL overexpression (+Dox) or cholesterol supplementation for 3 days. Target gene expression in each sample was compared to that in RUES1 hESCs. The error bars indicate the averages ± SDs (*n* = 3). One‐way ANOVA tests with Dunnett's multiple comparison test were performed (***p < 0.001, **p < 0.01, *p < 0.05). C) Western blotting shows the upregulation of ZSCAN4 after inducing PODXL overexpression in RUES1‐EPSCs after treatment with doxycycline (+Dox) for 3 day in hEPSCs. +: Dox, 2 × 10^−6^ m; ++: Dox, 4 × 10^−6^ m. The error bars indicate the averages ± SEMs (*n* = 3). P‐values were determined with one‐way ANOVA (***p < 0.001, **p < 0.01, *p < 0.05) with Dunnett's multiple comparisons test performed relative to −Dox EPSCs of target proteins. D) Representative images of hEPSCs and hESCs (RUES1 and RUES2) for P3 cells after TSC induction. CT27 and bTS11 are TSC‐positive controls.^[^ ^22^ ^]^ CT27: TSCs derived from a trimester placenta, bTS11: TSCs derived from a blastocyst. E) qRT‐PCR analyses for trophoblast marker genes in primed RUES2 hESCs, RUES2‐EPSCs −Dox, and RUES2‐EPSCs +Dox cultured in hTSC medium were measured. Relative gene expression levels were compared to primed RUES2 hESCs cultured in TSC medium. Error bars indicate averages ±SD (*n* = 3). P‐values were determined with an unpaired Student's t‐test (***p < 0.01, **p < 0.01, *p < 0.05) relative to the corresponding −Dox control group. F) Teratomas generated from engrafted cells (RUES1‐hESCs, RUES1‐EPSC −Dox, RUES1‐EPSC +Dox) by subcutaneous injection. H&E staining and immunohistochemistry analyses for four germ layers in teratomas are shown as indicated.

To functionally determine whether PODXL led to an extended pluripotency state, we generated Dox‐inducible PODXL expression in hEPSC lines (**Figure** **5**A). A closer examination of mRNAs revealed upregulation of several cleavage‐specific genes including *ZSCAN4*, *ARGFX*, *CPHX1*, *DPRX*, *DUXA*, *DUXB*, *LEUTX*, and the naive‐specific lncRNA *HERVK*, but downregulation of primed‐specific lncRNA, *HERVH* after induction of PODXL overexpression and cholesterol supplementation in hEPSCs (Figure 5B). We also found that the totipotent marker ZSCAN4 protein was upregulated after inducing PODXL overexpression and cholesterol supplementation (Figure 5C). This result leads us to hypothesize that PODXL may promote a cellular state close to totipotency which would have the potential to differentiate into ectoderm, endoderm, mesoderm, and trophectoderm. A distinguishing feature of totipotent‐like cells is the ability to differentiate into extra‐embryonic trophectoderm (TE) lineage like trophoblast stem cells (TSCs), where, for example, EPSCs have been suggested to differentiate into TSCs.^[^ ^22^ ^]^ In our experiments, both untreated control and Dox‐treated (PODXL‐overexpressing) hEPSCs were able to differentiate into expanded cobblestone‐shape colonies reminiscent of hTSCs (CT27, derived from the early placenta; bTS11, derived from blastocysts)^[^ ^23^ ^]^ in two passages (P2), while Dox‐treated hEPSCs exhibited higher expression of TSC markers (Figure 5D,E and Figure S5A, Supporting Information). In addition, both Dox‐treated or untreated control hEPSCs led to well‐differentiated teratomas with three embryonic germ layers and the immunohistological features of human beta‐chorionic gonadotropin (hCG) positive staining and hemorrhage areas, whereas hCG positive staining was not observed in teratomas derived from primed hESCs (Figure 5F and Figure S5B, Supporting Information). In contrast, tumor weight was significantly decreased after inducing PODXL knockout in hEPSCs (Figure S5C, Supporting Information). These data further support the importance of PODXL for pluripotency potential.

### 2.6 Transcriptional Landscapes and Dynamics of hEPSCs Overexpressing PODXL during Human–Mouse Chimera Development

**Figure 6:**
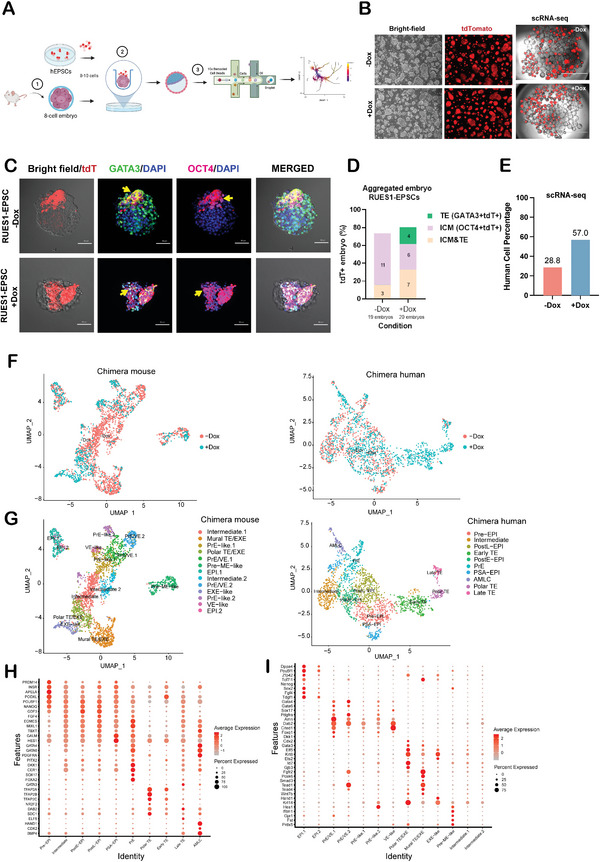
Inducing PODXL overexpression increases the developmental capability of human–mouse chimeras. A) Schematic of the generation and analyses of chimeric embryos derived from 8‐cell embryos aggregated with tdTomato (tdT)‐expressing RUES1‐hEPSCs. (Created with BioRender.com) B) Bright‐field images of RUES1‐hEPSC −Dox and hEPSC +Dox cells after 3 days. 8–10 hEPSCs were aggregated with an 8‐cell embryo and developed for ~37 h equivalent to the late blastocyst stage (E4.5‐E5.0) for scRNA‐seq analyses. C,D) Immunofluorescence staining identification and quantification of pre‐implantation chimeric embryos using the aggregated method with RUES1‐EPSCs. The percentage of E4.5‐E5.0 human–mouse chimeric embryos with different lineage contributions (ICM, TE, ICM and TE), stained for OCT4 (ICM), GATA3 (TE). tTd, tdTomato. Scale bar, 50 µm E) Bar plot showing the distribution of human RUES1‐EPSCs of the chimeric embryos. F) The UMAP plot of cells from human RUES1‐EPSCs and mouse cells in chimeric embryos, separately. Cells were colored by different experimental conditions (−Dox or +Dox). G) UMAP plot of cell clusters from human RUES1‐EPSCs and mouse cells in chimeric embryos, separately. Cell populations were labeled with numbers and different colors. EPI, epiblasts. PostE‐EPI, postimplantation early EPI‐like cells. PostL‐EPI, post‐implantation late EPI‐like cells. TE, trophectoderm. PrE, primitive endoderm. PSA‐EPI, primitive‐streak associated epiblasts. AMLC, amnion‐like cells. EXE, extra‐embryonic ectoderm. VE, visceral endoderm. pre‐ME, pre‐mesendoderm. H) Bubble plot of the expression of lineage‐specific marker genes from each cell cluster in human RUES1‐EPSCs and mouse cells in chimeric embryos.

The chimera assay is widely accepted as a gold standard for pluripotency/totipotency based on its capacity to test donor cell lineage potential in vivo. To evaluate PODXL's role in the chimeric competency of hEPSCs, we generated Tet‐on‐PODXL hEPSC lines that stably expressed tdTomato (tdT) fluorescence proteins (**Figure** **6**A,B). We generated human–mouse chimeric embryos by incorporating one 8C embryo with ~8‐10 cells of Dox‐treated or untreated control hEPSCs using an aggregation method. The embryos were cultured in media removing doxycycline for an additional ~37 h to grow further into the late blastocyst stage. The proliferation rate of tdT‐positive hEPSCs in blastocysts was noticeable. Approximately 80% tdT+ hEPSCs were detected in all mouse embryos (Figure 6B). We investigated the chimeric contribution of hEPSCs into embryonic (ICM, OCT4+) and trophectoderm (TE, GATA3+) and extra‐embryonic tissue (ExEm, CDX2+) lineages by immunofluorescence (IF). At the peri‐implantation stage, on average, by the aggregation chimera assay, the +Dox condition formed a higher percentage of ICM and TE (OCT4+& GATA3+) (35.0%) as compared with the −Dox condition (15.7%) (Figure 6C,D), while +Dox hEPSCs consisted of 59–82% of Em and ExEm (OCT4+& CDX2+), which was higher compared to untreated control hEPSCs (25–30%) (Figure S6A,B, Supporting Information). Using an embryo microinjection method to generate chimeras also showed that Dox‐treated hEPSC improved the chimera formation efficiency of hEPSCs (Figure S6C–E, Supporting Information).

To further explore the developmental trajectories of human–mouse chimeric embryos, scRNA‐seq analyses were carried out to profile the transcriptomes of human and mouse cells at the peri‐implantation embryo stage. The sequence reads in the untreated control (‐Dox) and Dox‐treated (+Dox) groups mapped to humans were confirmed with tdTomato and BSDWPRE sequences (Figure S6F, Supporting Information). After stringent filtering, 2015 human cells (‐Dox: 911, +Dox: 1104) and 3085 mouse cells (‐Dox: 2252, +Dox: 833) were used for further analyses. Human cells were present at a level of 57% in +Dox conditions, which was higher than 28.8% of human cells in the −Dox conditions, corroborating the finding that inducing PODXL overexpression enhances the chimera rate (Figure 6E). We used the default pre‐processing workflow of scRNA‐seq data by using the Seurat (v3.2.1) package and we clustered the scRNA‐seq dataset and found markers that define clusters of different cell types via differential expression analyses (Figure 6F,G). The UMAP of these cell types in the chimeric mouse embryos showed differences between +Dox and −Dox conditions, suggesting the host embryos were influenced by the incorporation of PODXL‐overexpressing hEPSCs. Based on the expression of known lineage‐specific markers, we identified 13 groups in chimeric mice and 10 clusters in chimeric human samples, and these representative lineage‐specific markers were employed for the creation of each cluster (Figure 6H,I). For example, the chimeric human cells in the Pre‐EPI‐like cluster expressed *POU5F1*, *NANOG*, *PRDM14*, *INSR*, *APELA*, and *PODXL*, while human cells in the PostE‐EPI‐like cluster co‐expressed *GDF3*, *FGF4*, *POU5F1* and *NANOG* (Figure 6H). Additionally, *FGF4*, *EOMES*, *MIXL1*, and *TBXT* appeared along with the PostE‐EPI‐like expressed genes suggesting human cells entered into a later stage of epiblast embryonic lineage differentiation‐like state (PostL‐EPI‐like). *GATA6*, *SOX17*, *GATA4*, and *PDGFRA* were expressed in the chimeric human cells and represented primitive endoderm‐like (PrE‐like or hypoblast) clusters (Figure 6H). We also observed that the chimeric human cells expressed high levels of *TFAP2A*, *TFAP2B*, and *TFAP2C* which are known trophoblast markers and, thus, were classified as trophectoderm‐like cells (Figure 6H). NR2F2 marks the maturation of the trophectoderm. NR2F2 expression was first detected in polar TE in late blastocysts. Then the expression is known to extend to all TE cells after implantation.^[^ ^24^ ^]^ We also identified populations specifically expressing *NR2F2* as a Polar TE‐like cluster (Figure 6H). Cells dominantly expressing levels of *GATA3* and relatively high levels of *ELF5* and *DAB2* were identified in a Late TE‐like cluster (Figure 6H). Cells expressing *BMP4*, *CDX2*, and *HAND1* were identified (Figure 6H), which have been reported as markers of extraembryonic mesoderm in nonhuman primates.^[^ ^25^ ^]^ Thus, this cluster of cells may undergo a cell fate transition into amniotic‐like cells (AMLC).

To assess the distribution of cells from different origins, for mouse cells in the chimeric embryos there were increasing percentages of cells enriched in clusters of Polar TE/EXE, EXE‐like, VE‐like, PrE‐like.2 cells in +Dox condition, whereas Mural TE/EXE, PrE‐like.1, PrE/VE.2, Intermediate.1, Intermediate.2 cells were enriched in the −Dox condition (Figure S6G, Supporting Information). As for human cells in the chimeric embryos, we also observed in the +Dox condition an increase in the cell percentage of Early TE‐like and Polar TE‐like cells (Figure S6G, Supporting Information). The expression of trophectoderm markers *SDC1*, *TFAP2A*, *TFAP2C*, *NRF2F2*, *CDX2*, and *GATA3* were increased in +Dox cells (Figure S6H, Supporting Information).

Interestingly, we observed that cholesterol supplementation increased the chimera rate in the ICM and TE lineages (OCT4+& GATA3+) (43%), and ICM lineages (OCT4+) (56.5%), while the control ICM and TE lineages of OCT4+& GATA3+ was 30.7%, and ICM lineages of OCT4+ was 38.4%. These results further support the importance of cholesterol signals in interspecies chimera formation (Figure S6I, Supporting Information).

Since proper cell fate specification and differentiation are critical for embryonic development, we examined the developmental trajectory of human cells and mouse cells in chimeras. We observed distinct trajectories between the −Dox and +Dox conditions for both mouse and human cells (Figure S7A,B, Supporting Information). In the chimeric mouse cells, the +Dox condition and −Dox condition showed similar pseudotime patterns during chimera development. However, in the chimeric human cells, the +Dox condition displayed an extended differentiated state as compared to −Dox human cells, suggesting the ability to promote proper differentiation of hEPSCs induced by PODXL overexpression (Figure S7B, Supporting Information). For both mouse and human cells, we performed lineage‐specific correlation analyses and observed that all chimeric lineage cells showed high correlation coefficients (> 0.7) compared to their counterparts in primary embryos in both the −Dox and +Dox conditions (Figure S7C, Supporting Information). Although human chimeric TE‐like cells were not completely identical to the wild‐type human embryo cells, the correlation coefficient was still high (both > 0.7). This suggests our observed chimeric embryo development is similar to that which occurs in wild‐type embryos.

### 2.7 Mutual Cell–Cell Interactions within Human–Mouse Chimeras after Inducing PODXL Overexpression

**Figure 7:**
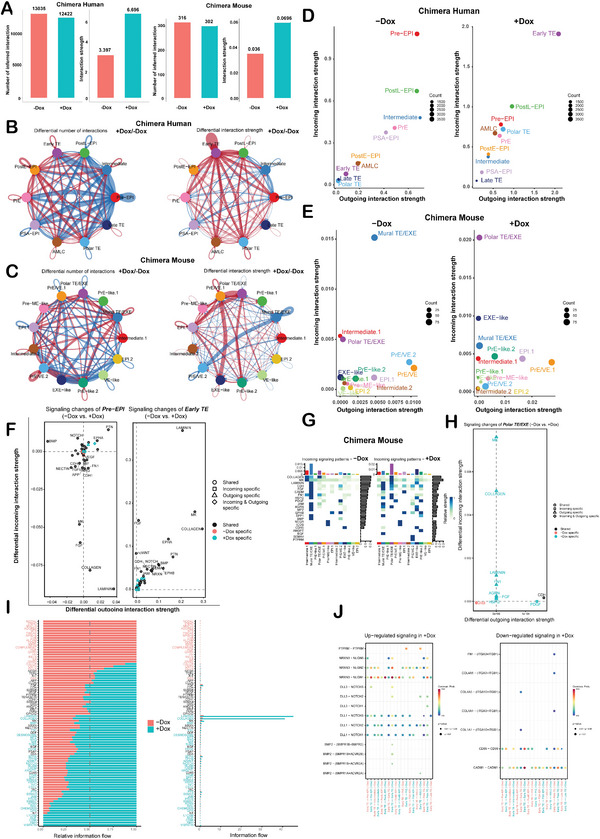
Cell–cell communication analyses of human–mouse chimeric embryos reveal that laminin and collagen signaling‐related pathways are dominant in PODXL overexpressing cells. A) The total number of interactions and interaction strength were displayed as bar plots showing the chimeric human RUES1‐EPSCs and the chimeric mouse embryos. B,C) In the cell–cell communication networks, circle plots of the number of interactions or interaction strength between −Dox and +Dox conditions are shown. Red (or blue) colored edges represented increased (or decreased) signaling in the +Dox compared to the −Dox conditions in the chimeric human RUES1‐EPSCs or the chimeric mouse embryos, respectively. D,E) Scatter plots showing significant changes in “outgoing” or “incoming” signals between −Dox and + Dox chimera human RUES1‐EPSCs or between −Dox and +Dox chimera mouse cells, respectively. F) Scatter plot showing the differential signals that change from −Dox to +Dox in the Pre‐EPI cell population and Early TE‐like cell population in the chimeric human cells. Positive values indicated the increase in the +Dox chimera human RUES1‐EPSCs while negative values indicated the increase in the −Dox chimeric human cells. G) Heatmap plot showing the “incoming” signals in different cell populations between −Dox and +Dox chimeric mouse cells. H) Scatter plot showing the signal changes from −Dox to +Dox and associated with Polar TE/ExE populations in the chimeric mouse cells. Positive values indicated the increase in the +Dox chimera mouse cells while negative values indicated the increase in the −Dox chimeric mouse cells. I) Significant signaling pathways ranked based on differences in the overall information flow within the inferred networks between −Dox and +Dox in the chimeric human RUES1‐EPSCs (right) and the information flow as converted into a 1.0 scale (left). The top signaling pathways colored red were enriched in the −Dox condition and those colored blue were enriched in the +Dox condition. The right panel showed collagen and laminin which were upregulated in +Dox cells shown by information flow (please refer to method details). J) Bubble plot showing the significant context‐specific signals upregulated or downregulated from the Early TE‐like cell population to other cell clusters in the chimeric human RUES1‐EPSCs.

To further delineate the crosstalk between mouse and human cells after PODXL overexpression at the peri‐implantation stage, we utilized gene expression data from cells as input. We modeled the probability of cell–cell communication via CellChat analyses.^[^ ^26^ ^]^ To compare the total cell communication numbers between −Dox and +Dox conditions, both the chimeric human and chimeric mouse cells showed similar results but displayed an increase in the interaction strength in the +Dox condition (**Figure** **7**A). These analyses indicate that PODXL overexpression can promote cell–cell communication in both the chimeric mouse and chimeric human cells. As displayed by a circle plot, we observed that Pre‐EPI‐like cells decreased the number of cell–cell communication interactions and Early TE‐like cells increased cell–cell communication interactions between all other cell clusters after PODXL overexpression in the chimeric human cells (Figure 7B). As for the chimeric mouse cells, both interaction number and strength in cell–cell communication were decreased in Mural TE/EXE after PODXL overexpression (Figure 7C). 2D scatter plots were generated to highlight the major “outgoing” (ligand) and “incoming” (receptor) cell–cell communication directions within the populations of the cells. Pre‐EPI‐like cells and Early TE‐like cells emerged as both “outgoing” sources and “incoming” targets in cell–cell communication in −Dox and +Dox conditions of human cells, respectively (Figure 7D). While in the chimeric mouse cells, Mural TE/EXE and Polar TE/EXE were the major “incoming” targets of cell–cell communication in −Dox and +Dox conditions, respectively (Figure 7E), while the major “outgoing” sources of cell–cell communication in −Dox and +Dox conditions of the chimeric mouse cells were the PrE/VE.1 cells (Figure 7E). In the chimeric human cells, the +Dox Early TE‐like cell population enriched pathways were LAMININ, COLLAGEN, MK, EPHA, BMP, and SEMA6 (+Dox specific) (Figure 7F). Interestingly, for the chimeric mouse cells in the Polar TE/EXE population, “incoming” pathways (receptors) such as COLLAGEN, MK, LAMININ, FN1, and AGRN showed upregulation in +Dox cells (Figure 7G,H). By comparing the direction of information flow, which is defined by the sum of the communication probabilities with all of the kinds of inferred networks among cells (i.e., the total weights in the network), we found that LAMININ and COLLAGEN signaling were predominant and shared among all clusters in comparison between the human −Dox and +Dox conditions (Figure 7I). For condition‐specific signaling pathways in the chimeric human cells, NODAL, ENHO, and MHCII were specific signals in −Dox condition, whereas VISFATIN, CD96, NGF, and L1CAM were specific signals in +Dox condition for all cell populations (Figure 7I). Since we found that Early TE‐like cells were dominant in the +Dox chimeras, we wanted to know what the signal was in Early TE‐like cells interacting with other cell clusters. Toward this end, we extracted the ligand‐receptor pairs with upregulated ligands and downregulated ligands in the +Dox condition. Cell–cell communication pathways of NRXN (cell–cell adhesion), PTPRM (cell–cell adhesion), NOTCH (cell–cell adhesion), and BMP2 (secreted pathway) were found upregulated in Early TE‐like cells, while pathways of CADM1 (cell–cell adhesion), CD99 (cell–cell adhesion) and FN1 (ECM‐receptor) were downregulated in Early TE‐like cells (Figure 7J). These data suggest that PODXL‐overexpressing hEPSCs may coordinate with host mouse cells, especially the Polar TE/EXE, which also upregulated similar pathways, to promote chimera formation.

## 3 Discussion

Limited data are available on the relationship between cholesterol metabolism and renewal in PSCs. It has been shown simvastatin impaired mouse ESC self‐renewal by regulating RhoA/ROCK‐dependent signaling in a cholesterol‐independent manner.^[^ ^27^ ^]^ In our data, blockade of the cholesterol pathway by simvastatin and AY9944 or depletion of cholesterol by MBCD severely impacted the self‐renewal ability of hPSCs relative to adult stem cells and fibroblasts (Figure 3H,I). PODXL recruited the RAC1/CDC42/actin network to regulate SREBP1 and SREBP2 maturation and lipid raft dynamics (Figure 4 and Figure S4G–L, Supporting Information) In brief, we found cholesterol is critical for PSC/EPSC self‐renewal and PODXL can regulate cholesterol biosynthesis through the RAC1/CDC42/actin network to further both regulate SREBPs in a SCAP‐dependent pathway and lipid raft formation. However, there is a paper reporting that statins are toxic only to karyotypically abnormal hESCs and not karyotypically normal hESCs.^[^ ^28^ ^]^ Notably, these cells were cultured with a high concentration of bFGF (16 ng mL^−1^) in the presence of 20% KSR, which contains high levels of cholesterol.^[^ ^29^ ^]^ By contrast, our cells were cultured in the chemically defined E8 medium, which is currently a widely used serum‐free and cholesterol‐free medium in stem cell research. Our HUES6 and H9 cells have normal karyotypes (Figure S3M, Supporting Information). Therefore, we suggest that the discrepancy between our results and the previous report is due to the differences in the cultural media. Cholesterol in KSR or high levels of bFGF may alleviate the toxicity of statins in karyotype normal cells. Notably, PODXL knockdown blocked self‐renewal in EPSCs cultured without KSR (Figure 2H) but this was not the case in a medium with 5% KSR (~4.4 µg mL^−1^ cholesterol),^[^ ^29b^ ^]^ which may have occurred because cholesterol is downstream of the PODXL pathway and could have prevented the deleterious effects of PODXL knockdown.

We found that inducing PODXL overexpression can enhance ZSCAN4 gene expression (Figure 5B,C), a totipotent marker, and promote trophoblast stem cell (TSC)‐like differentiation in vitro (Figure 5D,E, and Figure S5A, Supporting Information). These results are consistent with a previous report showing that human LCDM‐EPSCs can differentiate into hTSCs akin to day 8 trophoblasts and further differentiate into TE‐like derivatives under proper induction.^[^ ^22^ ^]^ In vivo experiments using hPSCs to generate human‐animal chimeras provide a platform to study early human embryogenesis and potentially regenerate human tissues and organs for replacement therapies. To our knowledge, we are the first to dissect the transcriptome landscape and differentiation trajectories of human–mouse chimeras at the peri‐implantation embryo stage by using scRNA‐seq. We found that transiently forcing PODXL overexpression in hEPSCs enhanced human–mouse chimerism from 28.8% to 57% (sc‐RNASeq) (Figure 6E). We also identified differentiated TE‐like cell populations within the human–mouse chimeras (Figure 6), which was similar to a previous study that discovered the TE‐like contribution of LCDM‐EPSCs in a chimera assay.^[^ ^4a^ ^]^ Although human LCDM‐EPSCs can specify epiblasts and primitive endoderm (or hypoblast), TE‐like cells were observed to be partially specified in a blastoid‐forming assay but expressing amnion markers.^[^ ^30^ ^]^ One report indicated that hEPSCs rarely contribute to TE‐derivatives in human‐monkey chimeras.^[^ ^31^ ^]^ The discrepancy between our results as compared to those observed in human‐monkey chimera studies may be attributed to the timing of the mixing of the hEPSCs with the host embryos. The first lineage segregations of ICM and TE occurs on the onset of the morula stage (E3.0) in the mouse embryos but on day 5 in human embryos.^[^ ^32^ ^]^ The hEPSCs were microinjected into the monkey blastocyst at day post fertilization (d.p.f.) 6 at a time when EPI and TE cell fate decisions were already well‐determined. Thus, the observation of only a few and/or the absence of TE lineage derivatives of incorporated hEPSCs in monkey embryos is not a surprise. In this study, we systemically evaluated the ability of PODXL overexpression (+Dox) cells compared to untreated control hEPSC (‐Dox) cells to enter both embryonic‐like and extraembryonic‐like lineages (Figure 6, Figures S6 and S7, Supporting Information). However, Posfai et al. suggested that EPSCs cannot contribute to trophectoderm based on their chimera assay and single‐cell analyses of mouse blastoid data.^[^ ^30^ ^]^ The discrepancy between our results compared to theirs might be due to the EPSC conversion rate and passage numbers in the EPSC‐LCDM media. We passaged cells in EPSC medium 20 to 40 times, while Posfai et. al. used fewer passages (mouse cells converted in their hands were used at least for 5 or 30 passages). The stability of the potency state of EPSCs may exhibit a great impact under different experimental methods. hEPSCs do enhance trophectoderm contribution, and transient PODXL overexpressing cells further enhance this phenomenon (Figure 5, Figure S5, Supporting Information; Figure 6, Figure S6, Supporting Information). However, incomplete conversion of trophectoderm in the chimera was observed, including lower expression levels of *GATA3* and co‐expression of *GATA3* and *CDX2* in Early TE and incomplete downregulation of *OCT4* in pan TE‐like cell populations (Figure 6H). This suggests that the chimera embryos may not be able to differentiate normally, and the EPSC condition needs to be improved further to reach the extended pluripotency state.

Interestingly, the reason why primed ESCs cannot form chimeras with mouse embryos is that primed hPSCs grow a lot slower than mouse ESCs.^[^ ^33^ ^]^ In our study, we found a relatively high human–mouse chimera efficiency in +Dox conditions in the peri‐implantation stage (57%) (Figure 6E), suggesting relatively fast cell growth and anti‐apoptosis properties of PODXL as an advantage for the formation of interspecies chimeras. Additionally, we also observed that cholesterol supplementation increased chimera rates in the ICM and TE lineages (OCT4+& GATA3+) (43%), and ICM lineages (OCT4+) (56.5%), which further supports the idea that PODXL and cholesterol signals enhance interspecies chimera formation (Figure S6I, Supporting Information).

Conserved cell–cell communication pathways were observed to be upregulated in COLLAGEN, LAMININ, and MK after inducing PODXL overexpression (+Dox) in both the chimeric human and chimeric mouse cells, implying PODXL's signaling role in the donor cells also expanded its effects onto the host mouse cells via cell–cell communication (Figure 7G and Figure 7I). At the single‐cell level, we found that the Early TE‐like cell populations act as a major signaling source and receiver to influence other clustered populations, with upregulated pathways including NRXN (NRXN3‐NLGN1/2/3), PTPRM (PTPRM‐PTPRM), NOTCH (DLL1/3‐NOTCHUES5/2/3) and BMP2 (BMPR1B‐ACVR2A/B) (Figure 7J). Based on these data, we anticipate that optimization of cell–cell contacts is critical for reducing the xenogeneic barrier in the interspecies chimeras for future studies.

In summary, we systemically discovered a monoclonal antibody against a specific glycosylated form of PODXL protein which has shown ihighly enriched in hEPSCs and hPSCs. Our new findings establish the importance of the molecular and cellular roles of PODXL in regulating cholesterol production, lipid rafts, and the actin cytoskeleton for the promotion of proper cell–cell communication events. Importantly, PODXL promotes integration between hEPSCs and host mouse embryo cells during early embryonic development in chimeras.

## 4 Experimental Section

### Primed hPSC Cultures

The HUES5 and HUES6 (S6) cell lines were kindly provided by Dr. Douglas A. Melton (Harvard University, Boston, MA, USA).^[^ ^34^ ^]^ The H9 (WA09) cell line was purchased from WiCells (Madison, WI, USA).^[^ ^1^ ^]^ The iPSC‐0207 cell lines were bought from the Food Industry Research and Development Institute in Taiwan. The inducible CRISPRn iPSC line (CRISPRn Gen1C) was purchased from Dr. Bruce R. Conklin's laboratory.^[^ ^18^ ^]^ Primed hPSCs were cultured under 20% O_2_ and 5% CO_2_ at 37 °C on mitomycin C‐inactivated mouse embryonic fibroblasts (MEF) that served as feeder cells. The hPSC medium was prepared as follows: DMEM/F12 was supplemented with 20% KSR, 1% GlutaMAX, 1% nonessential amino acids, 0.1 × 10^−3^ m ß‐mercaptoethanol (Sigma, M3148), and 8 ng mL^−1^ bFGF. For routine passage, primed hPSCs were detached with 0.1% collagenase IV every 5–7 days. The inducible CRISPRn Gen1C cell line was maintained in a chemically defined medium (E8 medium) supplemented with 0.5 µg mL^−1^ puromycin and 10 × 10^−6^ m ROCK inhibitor (ROCKi, LC Laboratories, Y‐5301) in feeder‐free conditions on Matrigel‐coated plates and subcultured by treatment with Accutase every 3–4 days. For feeder‐free conditions used for viral transduction, at room temperature, hPSCs were treated with Accutase for 3 min. Then the cells were washed two times with 1× PBS to remove MEFs. Cells were then suspended in a chemically defined medium (E8 medium) without ROCK inhibitor. The use of hESCs/hPSCs compiled with the IRB on Biomedical Science Research, Academia Sinica (AS‐IRB‐BM‐14019 and 17019), and biosafety rules (BSF0417‐00003640) in Taiwan.

### Human EPSC Cultures

hEPSCs were maintained in an N2B27‐LCDM medium under 20% O_2_ and 5% CO_2_ at 37 °C. A total of 500 mL of N2B27‐LCDM medium was prepared, containing 241 mL of DMEM/F12 (Thermo Fisher Scientific, 11330‐032), 241 mL of Neurobasal medium (Thermo Fisher Scientific, 21103‐049), 2.5 mL of N2 supplement (Thermo Fisher Scientific, 17502‐048), 5 mL of B27 supplement (Thermo Fisher Scientific, 12587‐010), 1% nonessential amino acids (Thermo Fisher Scientific, 11140‐050), 0.1 × 10^−3^ m *β*‐mercaptoethanol (Sigma, M3148), 1% GlutaMAX (Thermo Fisher Scientific, 35050–061), and 5% KnockOut serum replacement (optional) (Thermo Fisher Scientific, A3181502). We added recombinant human LIF (10 ng mL^−1^; Peprotech, 300–05), CHIR 99021 (1 × 10^−6^ m; LC Laboratories, C‐6556), IWR‐1‐endo (1 × 10^−6^ m; Abmole, M2782), DiM (2 × 10^−6^ m; Tocris, 1425) and MiH (2 × 10^−6^ m; Tocris, 3268), Y‐27632 (2 × 10^−6^ m; LC Laboratories, Y‐5301) to the N2B27 medium. hEPSCs were cultured on MEF feeder cells and passaged with 0.05% trypsin for 3 min at 37 °C.

### Cell Cultures

HEK‐293T cells and the fibroblast lines, CRL‐2097 and IMR‐90, were cultured in 10% DMEM (Thermo Fisher Scientific, 11965084), 1% GlutaMAX (Thermo Fisher Scientific, 35050–061), and 1% nonessential amino acids (Thermo Fisher Scientific, 11140‐050). The BJ‐5Ta cell line was cultured in a 4:1 mixture of DMEM and Medium 199 supplemented as follows: 0.01 mg mL^−1^ hygromycin B, 10% fetal bovine serum (FBS), 1% GlutaMAX (Thermo Fisher Scientific, 35050–061) and 1% nonessential amino acids (Thermo Fisher Scientific, 11140‐050). BMMSCs were maintained in reduced serum (2%) medium, MesenPRO RS Medium (Thermo Fisher Scientific, 12746012), and subcultured by TrypLE Express Enzyme (1X) (Thermo Fisher Scientific, 12605010) for 5 min at 37 °C. NSCs were differentiated from H9 hESCs using a serum‐free medium, PSC Neural Induction Medium (Thermo Fisher Scientific, A1647801), according to the manufacture's protocol.

### Monoclonal Antibody Screening

Monoclonal antibodies against hESCs were generated and characterized as described previously.^[^ ^35^ ^]^ Female BALB/CJ mice (4‐6‐weeks‐old) were challenged with HUES5 hESCs intraperitoneally every 3 weeks for four times. Splenocytes from immunized mouse were collected on day 4 after the final immunization. With 50% polyethylene glycerol, the spleen cells were fused with NSI/1‐Ag4‐1 myeloma cells. The hybridoma cells were suspended in DMEM supplemented with hypoxanthine‐aminopterin‐thymidine (HAT) and hybridoma cloning factor and then plated onto 96‐well plates. 20 hybridoma clones were generated by limited dilution and preserved in liquid nitrogen. By immunizing the hybridoma cells, ascites were generated from pristine‐primed BALB/CJ mice. A Protein G Sepharose 4G gel was used to isolate the monoclonal antibodies.

### LC‐MS/MS Identification of Peptides from Proteins Targeted by the hESC‐mAb‐5‐22 Antibody

With the presence of protease inhibitor cocktail tablet (Roche Diagnostics, Indianapolis, IN, USA) in the lysis buffer [1% Nonidet P‐40 (NP‐40), 150 × 10^−3^ m NaCl, 50 × 10^−3^ m Tris‐HCl, pH 7.4], hESC5 cells were lysed. Then hESC‐mAb‐5‐22 (ESCAb 5–22) was conjugated with protein G sepharose (GE Healthcare Biosciences) and incubated with the supernatant. The elution buffer (0.2 M glycine, pH 2.5, 150 × 10^−3^ m NaCl, and 1% NP‐40) was used to elute proteins complexed with ESCAb 5–22. Neutralization was performed with 1 M Tris‐HCl, pH 9.1 buffer. The eluted samples were subjected to SDS‐PAGE. The bands of interest were cut from the gel, reduced with 50 × 10^−3^ m dithioerythritol (DTE) in 25 × 10^−3^ m ammonium bicarbonate (ABC) at pH 8.5 for 1 hour at 37 °C, and alkylated with 100 × 10^−3^ m iodoacetamide (IAA) in ABC for 1 hour at room temperature. After washing with 50% acetonitrile in ABC, the gel was soaked in 100% acetonitrile and incubated with 0.02 µg trypsin for 16 hours at 37 °C. The digested peptides were extracted with 50% acetonitrile in 5% TFA, and concentrated using a Concentrator (Eppendorf, Hamburg, Germany). The samples were subjected to LC‐MS/MS sequencing as performed by the Core Facility for Proteomics and Structural Biology Research at Academia Sinica.

### Glycan Microarray

To fabricate the microarrays, 200 kinds of 10 × 10^−3^ m glycans with a functional linker were suspended in the printing buffer (0.005% Tween 20 in 300 × 10^−3^ m phosphate buffer, pH 8.5). About 0.6 nL of glycans were spotted (BioDot; Cartesian Technologies) by a robotic pin (SMP3; TeleChem International) from 96‐well plates onto maleimide‐coated glass slides (ZeroBkg surface; Microsurfaces, Inc). The microarray was designed as 16 grids on one slide, with 10 columns × 5 rows in one grid with 2 glycan repeats. The glycan numbers, structures, and printing format are listed (Figure S1F, Supporting Information). Printed slides were allowed to react in an atmosphere of 80% humidity for one hour followed by desiccation overnight. These slides were stored at room temperature in a desiccator before use. Serum obtained from mice was diluted with 1% BSA/PBST buffer (PBST buffer: 0.05% Tween‐20 in PBS, pH 7.4). Superblock blocking buffer (Pierce) was used to block the glycan microarrays for 1 hour at 4 °C and washed with PBST buffer three times. The serum dilutions were then introduced to the glycan microarray and incubated at 4 °C for 1 hour. Excess serum antibodies were washed out and the microarrays were incubated individually with Alexa Fluor 647‐conjugated goat anti‐mouse IgG antibody as the secondary antibody at 4 °C in dark for 1 hour. The slides were then washed three times with PBST and scanned at 635 nm wavelength with a microarray fluorescence chip reader (GenePix 4300A; Molecular Devices Corporation) and scanned images were analyzed with GenePix Pro‐6.0 analysis software (Axon Instruments, Union City, CA, USA).

### Flow Cytometry

hESCs were digested with Accutase for 3 min and stained by the manufacturer's instructions (eBioscience, 88‐8005‐72). Cells were resuspended at a density of 5 × 10^5^ cells in 100 µL of 1× binding buffer. We added 2.5 µL of Annexin V‐FITC for 20 min at room temperature in the dark. 2.5 µL of the PI solution was mixed in the cells for 10 min (modified from the manufacturer's protocol), PBS was added up to a volume of 500 µL, and cells were immediately analyzed using a flow cytometer (BD FACSCalibur, BD Biosciences).

### Embryoid Body (EB) Formation

For EB formation, hESCs were detached with 0.1% collagenase IV dissolved in DMEM/F12 for approximately 30 min, and the cell clumps were cultured in a conventional hPSC medium without bFGF for 13 days.

### Alamar Blue Assays and Trypan Blue Exclusion Assays

hESCs were incubated in E8 medium (Thermo Fisher, A1517001) containing 10% Alamar blue (stock concentration: 10×) for 5 hours. The bioreduction activity was measured at wavelengths of 570 nm and 600 nm. For cell counting, cells were treated with Accutase and resuspended in a medium. The dissociated cells were mixed with an equal volume of 0.2% trypan blue and counted with a hemocytometer.

### Alkaline Phosphatase Activity and Staining Assays

ALP activity was tested by adding p‐nitrophenyl phosphate (pNPP) (N7653, Sigma) and incubating cells at 37 °C and within 5 min. Then the absorbance was detected at 405 nm. For ALP image staining, cells were first washed with 1× PBS, fixed with 4% formaldehyde for 3 min, and washed twice with 1× PBS. After fixation, cells were stained with freshly prepared ALP staining solution. Then, cells were washed with PBS. The ALP staining solution was prepared by dissolving 1 mL of 10% 2‐amino‐2‐methyl‐1,3‐propanediol (Sigma, A9754), 5 mg of naphthol (Sigma, N5000), and 5 mg of fast red violet B (Sigma, F1631) in 9 mL of ddH_2_O.

### Lentivirus Production and hESC Transductions

With some modifications, lentivirus was produced following a previous protocol.^[^ ^36^ ^]^ Briefly, the day before transfection, about 7.5 × 10^6^ HEK293T cells were seeded per 10 cm dish. Cells were separately transfected with 19.2 µg of plasmids containing PODXL cDNA cloned into the pLAS3w.Pbsd vector (National RNAi Core Facility, Taipei, Taiwan), shPODXL (short hairpin RNA targeting PODXL), shHMGCR (short hairpin RNA targeting HMGCR) (National RNAi Core Facility) (please refer Table S1, Supporting Information for the sequences), and the corresponding vector controls along with 15.6 µg of helper plasmids (pCMV‐dR8.91:pMD2.G = 10:1 (w/w)). After 24 hours, the medium was refreshed with a new medium containing 1% BSA. The supernatants were collected on the following 3 days, filtered through a 0.45 µm filter, and concentrated by ultracentrifugation. For lentiviral transduction, hESCs or hEPSCs were seeded on Matrigel‐coated plates (without the ROCK inhibitor) and incubated for 2 hours (for hESCs) or 18 hours (for hEPSCs) in the lentivirus‐containing supernatants with 8 µg mL^−1^ protamine sulfate. For shRNA transduction, the infection process was performed twice (at an M.O.I. of 10 each time). For PODXL overexpression, viral infection was performed one time (at an M.O.I. of 5). ALP activity and AB assays were assessed in shRNA‐treated hESCs after 6 days of shPODXL lentivirus transduction. ALP activity and AB assays were assessed in PODXL overexpressing hESCs after 5 days of lentivirus transduction. ALP activity and AB assays were assessed in shRNA‐treated hEPSCs after 7 days of shPODXL lentivirus transduction. ALP activity and AB assays were assessed in PODXL overexpressing hEPSCs after 8 days of lentivirus transduction. The primers used for cloning and the shRNA target sequences are listed in Supplementary Table S1.

### Human iPSC Generation by Somatic Cell Reprogramming

The lentiviral production of pRRL.PPT.SF.hOKSM.idTomato.preFRT, a gift from Dr. Axel Schambach,^[^ ^37^ ^]^ was performed using the vector co‐transfected with 15.6 µg of helper plasmids (psPAX2:pMD2.G = 10:1 (w/w)) in HEK‐293T cells. Primary human foreskin fibroblasts (ATCC, CRL‐2097) were co‐transduced with the pRRL.PPT.SF.hOKSM. idTomato.preFRT virus (5 M.O.I.) and the overexpression virus (GFP and PODXL) (5 M.O.I.) or shRNA (shRFP and shPODXL) virus (10 M.O.I). On day 1–3‐post‐transduction, cells were fed daily with an induction medium (DMEM supplemented with 10% FBS, 250 × 10^−6^ m sodium butyrate, and 50 µg mL^−1^ ascorbic acid). On day 4 post‐transduction, cells were split and re‐plated in Matrigel‐coated six‐well plates. Cells were fed with induction medium until day 6 and were then switched to a 1:1 mixture of induction medium and mTeSR1 (STEM CELL, 85850) supplemented with 250 × 10^−6^ m sodium butyrate and 50 µg mL^−1^ ascorbic acid. On day 7–20, transfected cells were fed daily with mTeSR1. For testing PODXL knockdown or PODXL overexpression effects on iPSC reprogramming, human foreskin fibroblasts were co‐transduced with shPODXL or PODXL overexpression lentivirus and a lentiviral vector encoding four transcription factors (OKSM), and the iPSC colonies were analyzed on post‐transduction day 20.

### Microarray and GO Term Analyses

A PODXL overexpression cDNA microarray (Affymetrix HG U133 Plus 2) (GSE127218) and a published microarray were analyzed according to the GeneSpring GX 11 workflow. Genes with a change in expression of greater than 2‐fold change (up‐regulated in PODXL‐overexpressing versus GFP control cells) or less than 0.5‐fold change (downregulated in PODXL‐overexpressing versus GFP control cells) were considered differentially expressed genes. GO term analyses were conducted with the DAVID functional annotation tool. A heatmap of differentially expressed genes in PODXL and GFP transductants that showed a positive or negative change of at least 2‐fold was displayed. Data were log2 transformed and normalized to the mean. The heatmap was generated by Cluster3.0 and Java TreeView.

### RNA Purification and Quantitative Real‐Time PCR (qRT‐PCR)

Total RNA was purified with TOOLSmart RNA Extractor (Biotools, DPT‐BD24) with DNase treated for 45 min at 42 °C and reverse‐transcribed with the SuperScript III First‐Strand Synthesis System (Invitrogen, 18080051). qRT‐PCR was conducted using KAPA SYBR FAST PCR Master Mix (KAPA Biosystems, KR0389) on a QuantStudio 5 Real‐Time PCR System (Thermo Fisher Scientific). The data were analyzed using the ΔΔCT method. GAPDH mRNA levels were used as the normalization control. The qRT‐PCR primers are shown in Supplementary Table S2.

### sgRNA Design and Cloning

The MIT CRISPR Design Tool (https://www.crispr.mit.edu) was used to design sgRNAs, and the highest‐ranking sgRNAs with the lowest number of predicted off‐target sites in the genome were selected. sgRNAs were targeted to the 5’UTR and intron 1 sequences at the PODXL locus, with a minimal number of off‐target sites in the genome. sgRNA1 (at position −205 from the transcription start site (TSS)) and sgRNA3 (at position +460 from the TSS) were used in this study. The Cas9 sgRNA vector (Addgene# 68463) contained two BbsI restriction sites. We ligated a pair of double‐stranded oligos containing the targeting sequences with the BbsI‐digested Cas9 sgRNA vector. For the guide sequence of individual sgRNAs, please refer to Supplementary Table S3. The sgRNA efficiency was tested by co‐transfection of HEK293T cells with wild‐type Cas9, the sgRNA, and the surrogate reporter plasmid.

### Inducible CRISPR iPSC Line Production

The inducible iPSC lines were generated by Dr. Bruce R. Conklin's laboratory.^[^ ^18^ ^]^ A strong constitutive promoter (CAG) transcribes rtTA and is oriented in the opposite direction from the TRE3G element to prevent leaky expression of the transgene without doxycycline. CRISPRn Gen 1C iPSC lines were used in this study.^[^ ^18^ ^]^ Guide RNA sequences were cloned into the Cas9 sgRNA vector (Addgene, Plasmid #68463). iPSC lines were seeded at a density of 7 × 10^4^ cells per 24‐well plate with Rock inhibitor (10 × 10^−6^ m). After 24 hours, the Rock inhibitor was removed by replacing the medium with a fresh mTeSR1 medium without doxycycline (as the solvent control group) or with 2 × 10^−6^ m doxycycline for an additional 24 hours to induce Cas9 gene expression. Then, iPSC lines were co‐transfected with a pair of sgRNAs (sgRNA1/sgRNA3) and a vector containing a blasticidin expression cassette (pLAS3W‐eGFP‐Blasticidin) with TransIT‐LT1 Transfection Reagent (Mirus Bio, MIR 2304) for 24 hours. After transfection, the medium was replaced with an E8 medium, and cells were selected with 2.5 µg mL^−1^ blasticidin for 1 day. The medium was then changed every day with the medium that contained 5 µg mL^−1^ blasticidin with or without doxycycline for 3 to 5 days. Cell numbers were measured by Alamar Blue assays and pluripotency capacity was examined by measuring ALP activity.

### Genomic Deletion Assays

Inducible CRISPRn iPSCs were co‐transfected with individual pairs of sgRNAs, e.g., sgRNA1+sgRNA3, and pLAS3W‐GFP‐Blasticidin. Genomic DNA was extracted with DNA extraction kit (GG2002, Viogene) after 5 days of transfection. For genotyping PCR, 100 ng of genomic DNA in a 25 µL PCR mix was used with a KAPA HiFi HotStart PCR kit to amplify the targeted regions using primers flanking the targeted regions. The amplification primers used for genotyping PCR are listed in Supplementary Table S3.

### Western Blotting

Whole‐cell protein extracts were isolated from primed hPSCs or hEPSCs using RIPA lysis buffer (1% NP40, 50 × 10^−3^ m Tris (pH 8.0), 150 × 10^−3^ m NaCl, 2 × 10^−3^ m EDTA) supplemented with protease inhibitor cocktail (Roche, 04693132001). The protein concentration was determined by a Bio‐Rad Bradford Protein Assay Kit. The same amounts of protein were resolved by 10% SDS‐PAGE and transferred to 0.22‐µm PVDF membranes (Millipore, ISEQ00010). Membranes were incubated in 5% BSA/TBST at room temperature for 1 hour and then incubated with the appropriate antibodies in 5% BSA/TBST at 4 °C overnight. The primary antibodies in this study are: anti‐PODXL (1:1000; Santa Cruz, sc‐23904), anti‐c‐MYC (1:1000; Abcam, ab32072), anti‐TERT (1:1000; Abcam, ab183105), anti‐HMGCR (1:1000; Abcam, ab174830), anti‐SREBP1 (1:100; Santa Cruz, sc‐13551), anti‐SREBP1 (1:100; Santa Cruz, sc‐17755), anti‐SREBP2 (1:1000; Abcam, ab30682), anti‐OCT4 (1:1000; Cell Signaling Technology, 2890), anti‐KLF4 (1:1000; Abcam, ab72543), anti‐ZSCAN4 (1:1000; Gentex, GTX123211), anti‐FLOTILLIN‐1 (1:1000; BD Biosciences, 610821), anti‐ITGA2 (1:1000; Abcam, ab133557), anti‐GAPDH (1:5000; Abcam, ab9485), anti‐RAC1 (1:1000; Millipore, 05–389), anti‐CDC42 (1:500; Millipore, 05–542), anti‐*β*‐Tubulin (1:5000; Sigma, SAB4200715) and anti‐*β*‐Actin (1:5000; Sigma, A1978). Membranes were washed three times with TBS/0.2% Tween‐20 and incubated with the corresponding secondary antibodies at 4 °C overnight.: anti‐rabbit IgG, HRP‐linked antibody (1:10 000; Jackson Immuno Research, 711‐036‐150); anti‐mouse IgG, HRP‐linked antibody (1:10 000; Jackson Immuno Research, 711‐036‐152). After three washes in TBS/0.2% Tween‐20, immunoreactions were detected with ECL solution (Thermo Fisher Scientific, 34095). Quantifications of Western blots were performed using ImageJ using the “Analyze/Calibrate/Function‐Uncalibrated OD” and “Analyze/Gels function”.

### Cholesterol Inhibition Tests and Cholesterol Supplementation Rescue Assays

Cells were treated with various concentrations of cholesterol inhibitors (Simvastatin, AY9944, and MBCD) in a culture medium for 3 days and analyzed to measure the relative cell numbers (Alamar blue assays). IC_50_ values were calculated by using GraphPad Prism 5. In cholesterol rescue experiments, synthetic cholesterol (500× concentrate, 0.25% w/v, Sigma, #S5442), but not a cholesterol lipid concentrate (Thermo Fisher Scientific, 12531018), can rescue the PODXL knockdown phenotype. In brief, cholesterol was co‐treated at the second time of shPODXL lentivirus treatment at a final concentration of 2× cholesterol in E8 medium for the subsequent 5 days and then cells were subjected to Western blot analyses.

### Inhibitor Treatment of Potential Direct Downstream Effectors of PODXL

Inhibitors were selected, and cells were treated with them in conditions without inducing cell death in GFP‐ and PODXL‐overexpressing hESCs in E8 culture medium. These inhibitors included ZCL278 (CDC42 inhibitor, 50 × 10^−6^ m, 24 ho),^[^ ^38^ ^]^ EHop‐016 (Rac1 inhibitor, 10 × 10^−6^ m, 2 ho),^[^ ^39^ ^]^ and Latrunculin A (actin polymerization inhibitor, 0.4 × 10^−6^ m, 2 hours).^[^ ^40^ ^]^

### Co‐immunoprecipitations

The identification of PODXL interacting proteins was verified by co‐immunoprecipitation (Co‐IP) as described in a previous paper with some modifications.^[^ ^41^ ^]^ Cells were rinsed with 1 × PBS and excess 1 × PBS was removed by vacuum suction. Cells were lysed by immediately adding 0.5 mL of ice‐cold TNI lysis buffer (TNI buffer: 0.5% Igepal CA‐630, 50 × 10^−3^ m Tris (pH 7.5), 250 × 10^−3^ m NaCl, 1 × 10^−3^ m EDTA, and 1× Protease Inhibitor cocktail, 2 × 10^−3^ m Na_3_VO_4_, 2 × 10^−3^ m PMSF) per 1 × 10^7^ cells in a culture dish and incubated for 30 min on an orbital shaker at 4 °C. The cell lysate was scraped off with a cell scraper and transferred to a 1.5 mL microcentrifuge tube. The cell lysate was sonicated on a high‐power mode for 3 cycles (sonication cycle: 30 s ON, 30 s OFF) at 4 °C (Bioruptor Plus, Diagonde). A probe sonicator was not used which will lead to a loss of interactors. The cell lysate was centrifuged at 18000 × g for 30 min at 4 °C. 50 µL of Dynabeads Protein G (Thermo Fisher Scientific, 10004D) per Co‐IP reaction were precleaned and added to 1.5 mL centrifuge tubes and washed with 500 µL of washing buffer (10 × 10^−3^ m Tris‐HCl (pH 7.5), 0.5 × 10^−3^ m EGTA, 1% Triton X‐100, 0.1% SDS, 0.1% sodium deoxycholate, 140 × 10^−3^ m NaCl, 1 × Complete ULTRA EDTA‐free Protease Inhibitor cocktail, 2 × 10^−3^ m Na_3_VO_4_ and 2 × 10^−3^ m PMSF) 2 times by placing the tube on the magnetic tube stand to separate the beads from the solution and remove the supernatant. The crude cell lysate was precleaned with an equivalent amount of Dynabeads Protein G (Thermo Fisher Scientific, 10004D) used per Co‐IP reaction for 1 hour at 4 °C with head‐over‐head rotation. The antibody‐coupled Dynabeads Protein G was prepared by adding 10 µg of IgG control antibody (Biolegend, 400202), anti‐PODXL antibody (Thermo Fisher Scientific, 12‐8873‐42), or anti‐SCAP antibody (Thermo Fisher Scientific, A303‐554A) to the precleaned magnetic beads in a final 200 µL of IP washing buffer and incubated for 2 hours at 4 °C with gentle rotation. The pre‐cleaned cell lysate was collected into a new tube by placing the tube on the magnetic tube stand to separate the beads from the solution, the supernatant was removed, and the protein concentrations were measured. 2.5 mg of total protein cell lysate was added into antibody‐conjugated magnetic beads in a final 500 µL volume of TNI lysis buffer and incubated overnight with gentle rotation at 4 °C. The supernatant was removed, and magnetic beads were washed 3 times with 500 µL of TNI lysis buffer. 500 µL of TN lysis buffer (TNI buffer without Igepal CA‐630) was added and beads were transferred to a new 1.5 mL tube and beads were washed 2 times with TN lysis buffer. All the supernatant was carefully removed, and the beads were frozen at −80 °C overnight to increase yield in the following steps. Proteins were eluted with 40 µL of elution buffer (0.2 M glycine (pH 2.3), 0.5% Igepal CA‐630) for 40 min at 37 °C with shaking. Elute was neutralized with 1/10 vol (v/v) freshly prepared 1 M Na_2_CO_3_. The final 1× sample buffer was added to elute and boiled for 10 min, and the supernatant was ready for SDS‐PAGE analyses.

### RAC1/CDC42 Activation Pulldown Assays

Activities of RAC1 and CDC42 were detected using a Rac1/Cdc42 activation magnetic beads pulldown kit (Millipore, 17–10394) according to the manufacturer's instructions. Cells (~ 1 × 10^7^) were rinsed with ice‐cold 1 × PBS and 0.5 mL of ice‐cold MLB (1× MLB, 10 µg mL^−1^ aprotinin, 50 × 10^−3^ m NaF, 25 × 10^−3^ m *β*‐glycerol phosphate, 2 × 10^−3^ m Na_3_VO_4_, 10 µg mL^−1^ leupeptin, 2 × 10^−3^ m PMSF) to rinse cells in culture plates on ice. The cells were scrapped with a cell scraper on ice and the lysates were transferred to 1.5 mL microcentrifuge tubes and incubated on ice for 30 min. The insoluble cell debris was removed by centrifugation (14000 × g, 30 min, 4 °C) and supernatants were transferred to a new tube. Cell extracts were stored on ice for immediate use or snap‐frozen in liquid nitrogen and stored at −80 °C for long‐ term storage. Protein concentrations of cell extract were quantified, and aliquots were taken out as a sample input. For assays, 15 µL of the Rac1/Cdc42 assay reagent (PAK‐1 PBD magnetic beads) per 0.5 mL of cell extract (containing 4 mg to 7 mg of total protein) was added. The reaction mixtures were incubated for 1 hour at 4 °C with gentle agitation. The beads were pelleted by spinning down for a few seconds and the tubes were placed on a magnetic tube stand for 1 min. Then the supernatants were discarded. With 0.5 mL ice‐cold MLB buffer, the beads were washed 3 times (transferring the mixtures to a new 1.5 mL tube in a final wash step). The beads were resuspended in 40 µL of MLB with a final 1× sample buffer and boiled for 10 min. The supernatants were mixed, and the beads were subjected to Western blotting analyses. The gel loading volumes were judged according to equal protein amounts of the sample protein input levels for comparisons. PVDF membranes were incubated in 5% BSA/TBST at room temperature for 1 hour and then incubated with the appropriate antibodies in 5% BSA/TBST overnight at 4 °C. The antibodies are listed below: anti‐RAC1 (clone 23A8, 1:1000; Millipore, 05–389) and anti‐CDC42 (1:250; Millipore, 05–542). Membranes were then washed three times with TBS/0.2% Tween‐20 and incubated with the corresponding secondary antibodies (1:10 000) overnight at 4 °C. After three washes in TBS/0.2% Tween‐20, immunoreactions were detected with ECL solution (Thermo Fisher Scientific, 34095).

### Actin Polymerization Rescue Assay

iPSC lines were seeded at a density of 7×10^4^ cells per 24‐well plate with the Rock inhibitor (10 × 10^−6^ m). After 24 hours, the Rock inhibitor was removed by replacing the medium with fresh mTeSR1 medium without doxycycline (as the solvent control group) or with 2 × 10^−6^ m doxycycline for an additional 24 hours to induce Cas9 gene expression. Then, iPSC lines were co‐transfected with a pair of sgRNAs (sgRNA1/sgRNA3) and a vector containing a blasticidin expression cassette (pLAS3W‐eGFP‐Blasticidin) with TransIT‐LT1 Transfection Reagent (Mirus Bio, MIR 2304) for 24 hours. After transfection, the medium was changed into the E8 medium, and cells were selected with 2.5 µg mL^−1^ blasticidin for one day and supplied with 50 × 10^−9^ m Jasplakinolide (Jas) for another 5 days with 5 µg mL^−1^ blasticidin.

### Cholesterol Quantification

Cholesterol levels in whole‐cell lysates were calculated by an Amplex Red cholesterol assay (Invitrogen). Briefly, samples were diluted in reaction buffer, and an equivalent volume of Amplex Red working solution (300 × 10^−6^ m Amplex Red, 2 U mL^−1^ cholesterol oxidase, 2 U mL^−1^ cholesterol esterase, and 2 U mL^−1^ horseradish peroxidase) (Thermo Fisher Scientific, A12216) was added. Samples were incubated at 37 °C for 30 min, and the fluorescence was measured at EX:560 nm and Em:590 nm using a microplate reader. Cholesterol values were calculated using known cholesterol solutions and normalized to the protein content as measured by a Bradford Protein Assay Kit (Bio‐Rad).

### Metabolome Analysis

For PODXL‐overexpressing HUES6 samples, 1×10^7^ cells of each were collected after 4 days. Cell pellets were snap‐ frozen in lipid nitrogen for 1 minute and then stored at −80 °C for subsequent extraction. The metabolome analysis was performed by Metabolomics Core Facility at Agricultural Biotechnology Research Center, Academia Sinica. Metabolites and lipids were extracted following SIMPLEX with some modifications.^[^ ^42^ ^]^ 225 µL of cold MeOH were added to the cells and were vortexed for 20 s and incubated in lipid nitrogen for 1 minute. The samples were thawed at room temperature and then sonicated with high power mode for 10 cycles (sonication cycle: 30 s ON, 30 s OFF) at 4 °C (Bioruptor Plus, Diagonde). The cell lysis cycle and the sonication step were repeated two times. Then 750 µL of cold MTBE (Methyl‐tert‐butyl‐ether, Sigma‐Aldrich, 34875) were added, and the mixture was incubated for 1 hour at 4 °C under agitation. Phase separation was induced by adding 188 µL of water with 0.1% ammonium acetate. The extract was centrifuged at 10000 xg for 5 min, and the upper phase was collected, dried by SpecVec, and dissolved in 100 µL of cold MeOH for further analysis (lipid fraction). For protein precipitation, cold MeOH was added to the remaining lower phase in a final ratio of 4:1, v/v MeOH/H_2_O, and the samples were incubated for 1 hour at −20 °C, followed by centrifugation at 13000 xg for 12 min at 4 °C. The resulting supernatant was collected, dried by SpecVec, and dissolved in 100 µL of cold MeOH (Metabolite fraction). And the remaining protein pellet was dried and recorded as total cell amounts for normalization. Extracts were further centrifuged for 12 min (13000 xg) at 4 °C to remove any insoluble debris, and then stored at −20 °C before LC‐MS/MS analysis.

### Lipid Raft Isolation

Lipid raft extraction was examined with some modifications.^[^ ^43^ ^]^ Whole‐cell lysates were resuspended in buffer A (1% Brij 98, 25 × 10^−3^ m 2‐(N‐morpholino)‐ethanesulfonic acid, 150 × 10^−3^ m NaCl (pH 6.5), 2 × 10^−3^ m Na_3_VO_4_, 2 × 10^−3^ m PMSF, and protease inhibitor cocktail). After 30 min of incubation on ice, lysates were centrifuged at 15000 xg for 20 min, and the supernatants (containing the Brij 98‐soluble fraction) were collected as the non‐raft fractions. The insoluble pellets were resuspended in buffer B (1% Brij 98, 10 × 10^−3^ m Tris‐HCl (pH 7.6), 500 × 10^−3^ m NaCl, 15 × 10^−3^ m octyl‐*β*‐D‐glucopyranoside, 2 × 10^−3^ m Na_3_VO_4_, 2 × 10^−3^ m PMSF and protease inhibitor cocktail) for 30 min on ice. The buffer B supernatants were collected as the lipid raft fractions after 20 min of centrifugation at 15000 xg.

### Preparation of Detergent‐Resistant Membrane Fractions via Sucrose Gradient Ultracentrifugation

An alternative fractionation method for isolating lipid rafts was performed via centrifugal flotation through a sucrose gradient. EGFP‐overexpressing hESCs (2 × 10^7^) and PODXL‐overexpressing hESCs (4 × 10^7^) were harvested by Accutase treatment and washed with 1× PBS. 0.5 mL of ice‐cold TNE buffer was used to extract the cells. The TNE buffer contains 20 × 10^−3^ m Tris‐HCl (pH 8.5), 150 × 10^−3^ m NaCl, and 5 × 10^−3^ m EDTA) containing 1% Brij 98 (polyoxyethylene (20) oleyl ether; Sigma, P5641), 15 × 10^−3^ m octyl‐*β*‐D‐glucopyranoside (Sigma, O8001), protease inhibitor cocktail (Roche, 04693132001), 1 × 10^−3^ m Na_3_VO_4_ and 1 × 10^−3^ m PMSF. Next, lysates were passed 10 times through a 25‐gauge needle to disrupt the cells and were further incubated on ice for 1 hour. Then, 0.5 mL of ice‐cold 80% (w/v) sucrose was prepared in the lysis buffer. With a 5.2 mL polyallomer centrifuge tube (Beckman Coulter, 344057), the mixture was put at the bottom. Different density of sucrose solution was added in sequence, that is 30% (1.8 mL), 20% (0.8 mL), 10% (0.8 mL), and 5% (0.7 mL) sucrose in lysis buffer (with detergent). Finally, with 0.1 mL of lysis buffer. Samples were subjected to ultracentrifugation in a Beckman L90K ultracentrifuge (Fullerton, CA, USA) with a SW50Ti rotor for 20 hours (48,000 rpm) at 4 °C. Eight fractions (0.5 mL/fraction) were collected beginning at the top of the gradient. Proteins were separated by SDS‐PAGE and analyzed by immunoblotting with an equal volume of each fraction. For mass spectrometry (MS) analyses, proteins were precipitated from the selected fractions by the methanol/chloroform method. The protein pellets were then frozen at −80 °C.

### Protein Reduction and Alkylation

Protein extracts from the lipid raft‐enriched (fractions 2–3) and non‐raft membrane (fractions 7–8) fractions were dissolved in 4% SDS buffer (50 × 10^−3^ m Tris‐HCl (pH 7.4), 4% SDS, and 5 × 10^−3^ m EDTA) at room temperature for 10 min. Protein concentrations were evaluated by the Bradford method. Following dilution with 3 volumes of Dilution Buffer (50 × 10^−3^ m Tris‐HCl (pH 7.4), 150 × 10^−3^ m NaCl, 5 × 10^−3^ m EDTA, 0.2% Triton X‐100, 1 × 10^−3^ m PMSF, and protease inhibitor cocktail). 20 µg of protein were reduced with Reduction Buffer (10 × 10^−3^ m 1,4‐dithioerythritol (DTE), 8 M urea, and 25 × 10^−3^ m ammonium bicarbonate (ABC)) for 60 min at 37 °C and was then alkylated with 55 × 10^−3^ m iodoacetamide (IAM) in 25 × 10^−3^ m ABC for 35 min at room temperature in the dark. Then 5% SDS‐PAGE was used to separate the samples by running the gel at 80 V for 15 min. Each lane was cut into several equal volumes of cubed gel slices. The gel slices were transferred into a clean polypropylene (PP) microcentrifuge tube and destained. Proteins were subjected to in‐gel digestion with mass spectrometry grade Lys‐C in 25 × 10^−3^ m ABC (enzyme: protein = 1: 10) and incubated at 37 °C for 3 hours. An equal volume of mass spectrometry grade trypsin was added and incubated further at 37 °C for at least 16 hours. Digested peptides were successively extracted twice with 50% acetonitrile (ACN) and 5% trifluoroacetic acid (TFA) and the two extracted peptide solutions were combined and dried in a Speed Vac.

### Lipid Raft Immunofluorescence Staining

For lipid raft labeling, cells were treated with CTxB‐Alexa488 (1:100; Thermo Fisher, V34403) and anti‐PODXL antibody (1:50; R&D, MAB1658) in E8 medium (Thermo Fisher, A1517001) for 2 hours at 4 °C with gentle shaking. The medium was removed, and an anti‐CTXB antibody (1:200, Thermo Fisher, V34403) was added for lipid raft clustering at 4 °C for 20 min. To fix the cells, 4% paraformaldehyde (Sigma, P6148) was used at room temperature for 10 min. And then the cells were blocked with blocking solution (10% donkey serum, 0.5% Triton‐X100) at room temperature for 30 min. Secondary anti‐mouse Alexa647‐labeled IgG secondary antibody (Jackson ImmunoResearch, 715‐605‐150) was used to label PODXL via incubation at room temperature for 2 hours. Nuclei were stained with DAPI (0.5 µg mL^−1^, D9542) for 10 min. Cells were mounted with ProLong Diamond Antifade mounting reagent (Thermo Fisher Scientific, P36965).

### RAC1, PODXL, and Actin Confocal Imaging

HUES6 hESC transducted with PODXL overexpressing virus and selected for blasticidin for 4 days. Anti‐RAC1 (1:200; Millipore, 05–389), Anti‐PODXL (1:50; R&D, MAB1658) antibodies were added in E8 medium, and cells were live stained at 4 °C for 2 hours with gentle shaking. Cells were rinsed with 1 × PBS two times and fixed with 4% paraformaldehyde for 10 min. Cells were blocked for 30 min (10% donkey serum, 0.5% Triton‐X100 in 1 × PBS) and then exposed to Donkey anti‐Mouse IgG (H+L) Highly Cross‐Adsorbed Secondary Antibody, Alexa Fluor 488 (1:400; Invitrogen, A‐21202), Donkey anti‐Goat IgG (H+L) or Cross‐Adsorbed Secondary Antibody, Alexa Fluor 555 (1:400; Invitrogen, A‐21432) antibodies in staining buffer [5% donkey serum (Millipore, S30), 0.1% Triton‐X100 in 1 × PBS] at RT for 2 hours with gentle shaking. Cells were washed three times with washing buffer [2% donkey serum (Millipore, S30), 0.05% Triton‐X100 in 1 × PBS]. 5 µL of Alexa Fluor 647 Phalloidin (Thermo Fisher Scientific, A22287) methanolic stock solution was diluted into 300 µL of staining solution for each reaction to be stained at 4 °C for 2 hours with gentle horizontal shaking. Nuclei were stained with DAPI (0.5 µg mL^−1^, D9542) for 10 min. Cells were mounted with ProLong Diamond Antifade mounting reagent (Thermo Fisher Scientific, P36965).

### F‐Actin Staining

Cells were transducted with GFP‐ or PODXL‐overexpressing lentivirus for 5 days and then treated with RAC1 inhibitor (EHop‐016, 10 × 10^−6^ m, 2 hour), CDC42 inhibitor (ZCL278, 50 × 10^−6^ m, 24 hours) or actin inhibitor (Latrunculin A, 0.4 × 10^−6^ m, 2 hours), respectively. Cells were washed with 1 × PBS. 4% paraformaldehyde (Sigma, P6148) was used to fix the cells at room temperature for 10 min. Then the cells were blocked with a blocking solution (5% donkey serum; Millipore, S30) at room temperature for 30 min. 2.5 µL of Alexa Fluor 647 Phalloidin (Thermo Fisher Scientific, A22287) methanolic stock solution was diluted into 300 µL of staining solution (5% donkey serum, 0.1% Triton‐X100) for each reaction to be stained at 4 °C overnight with gentle horizontal shaking. Nuclei were stained with DAPI (0.5 µg mL^−1^, D9542) for 10 min. Cells were mounted with ProLong Diamond Antifade mounting reagent (Thermo Fisher Scientific, P36965).

### G‐actin/F‐Actin In Vivo Assays

The experimental procedure was followed according to the manufacturer's instructions (Cytoskeleton, Cat#BK037). To assay polymerized actin, GFP‐overexpressing hESCs (1 × 10^7^) and PODXL‐overexpressing hESCs (1 × 10^7^) were harvested by Accutase treatment 5 days after lentiviral transduction. Cells were lysed in 250 µL of warm LAS2 detergent‐based lysis buffer (1 mL of LAS1 buffer, 10 µL 100 × 10^−3^ m ATP stock, and 10 ul 100× protease inhibitor cocktail stock) and incubated at 37 °C in a water bath for 10 min. 250 µL cell lysate was centrifuged to pellet cell debris. Supernatants were collected for centrifugation at 37 °C for 1 hour. The G‐actin is soluble in detergent‐based lysis buffer and F‐actin will be pelleted at the bottom of the tube. The supernatant (G‐actin fraction) was removed, and the pellet (F‐actin fraction) was dissolved with 250 µL of F‐actin depolymerization buffer and incubated on ice for 1 hour. The samples were pipetted up and down or vortexed every 15 min to help F‐actin resuspension (5% SDS was added to promote resuspension) 50 µL of 5× protein sample buffer was added, and samples were boiled for 10 min. 10 µL of each sample was taken for SDS‐PAGE analyses. After protein transfer, the PVDF membrane was stained with Ponceau S solution (BIOTIUM, Cat#22001) to stain total protein at 4 °C overnight with gentle horizontal shaking. The membranes were rinsed with ddH_2_O, and bright field image quantification was performed. Membranes were washed with TBS/0.2% Tween‐20 several times and membranes were blocked with 5% Animal work was approved as described in the Western blot analyses section.

### In Vivo Teratoma Formation and Immunochemistry Assay

Approximately 3 × 10^6^ hEPSCs mixed with Matrigel (1:1 ratio) were injected subcutaneously into NOD.Cg‐Prkdcscid Il2rgtm1Wjl/SzJ (NSG mice) (*n* = 6 for each group). Doxycyclin (Dox) was dissolved in drinking water at a 1 mg mL^−1^ concentration along the experiment term. The animals were sacrificed after 7 weeks of cell injection. All tumor sizes did not exceed 1.5 cm in diameter. The teratomas were removed and weighed then fixed with 4% paraformaldehyde and embedded in paraffin and processed for hematoxylin and eosin staining. For IHC, hEPSC‐derived and primed hPSC‐derived teratomas by immunostaining with the antibody specific to Human alpha‐Fetoprotein (AFP) (R and D Systems, AF1369; 1:50), Human Smooth Muscle Actin antibody [1A4] (Agilent, M0851; 1:200), Tubulin beta 3 (TUBB3) antibody (TUJ1) (BioLegend, 801202; 1:1000) and human trophoblast marker hCG*β* [EPHCGR2] (Abcam, ab131170; 1:60) at 4 °C overnight. The slides were then stained with an HRP‐conjugated secondary antibody (Dako EnVision+ Dual Link System‐HRP (DAB+), Dako, K4065).

### Superovulation

ICR (breeding stock from BioLASCO Taiwan Co., Ltd.) was used in this study. Superovulation was performed using 8–10 weeks female mice i.p. treated with CARD HyperOva (Cosmo Bio Co., Ltd., KYD‐010‐EX‐X5) 7.5 IU and later up 48 hours i.p. treated with hCG (Sigma, CG‐10) 7.5 IU and collect embryos in the morning of E2.5. Male ICR used to mate is 8–20 weeks old. Laboratory mice stayed at 12 light/12 dark cycles (7:00–19:00). The study is compliant with all the relevant ethical regulations regarding animal research. Institutional Animal Care and Use Committee (IACUC) (17‐03‐1068) at Academia Sinica in Taiwan approved all the animal work.

### Interspecies Chimera Assay

To isolate eight‐cell‐stage embryos, embryos from female ICR (week 8–10, BioLASCO Taiwan Co., Ltd.) were collected on the morning of the day E2.5 by flushing the oviduct. The zona pellucida was removed using acid Tyrode's solution (Sigma‐Aldrich, T1788) briefly for 5–10 s, and the embryos were washed thoroughly in a prewarmed M2 medium. The denuded embryos were transferred to drops of N2B27‐LCDM (5% KSR, 10 × 10^−6^ m Y‐27632) for at least 4 hour before aggregation and then transferred to drops of G‐2TM PLUS (Vitrolife, 10132) media. Typsinized tdTomato‐labeling hEPSCs were suspended in N2B27‐LCDM (5% KSR, 10 × 10^−6^ m Y‐27632) for 30 min on ice. To generate aggregated chimeras, 8–10 single hEPSCs and one 8C embryo were brought together into the bottom of a microwell. In a plastic tissue culture dish (Falcon, 351007), the microwells were produced by an obtuse‐end needle that presses at the dish's bottom. Drops of media were placed under the oil. Such aggregation chimeras were grown in media removing doxycycline for additional ~37 hours for downstream analyses. For embryo microinjection, eight‐cell stage embryos were collected as previously described. About 8–10 single trypsinized hEPSCs were injected into one 8C embryo and the injected embryos were cultured in the N2B27‐LCDM medium for the first 4 hours (for the culture of chimeric embryos injected with hEPSCs, the addition of 10 × 10^−6^ m Y‐27632 is recommended), and then they were changed into the G‐2 PLUSTM media. For the cholesterol addition experiment, a final concentration of 2x cholesterol was added into N2B27‐LCDM (5% KSR) for 4 days of culturing. hEPSC colonies were lifted with 0.1% collagenase and digested into single cells by trypsin. These single cells were put on ice and ready for aggregation with 8C embryos.

### Immunofluorescence Staining of Chimeras

Whole‐mount immunofluorescence staining of E4.5‐E5.0 embryos was performed. In brief, embryos were washed in 1 × PBS containing 0.1% PVA. Then the embryos were treated with 4% paraformaldehyde (PFA) at room temperature for 30 min. Then the embryos were washed twice in 1× PBS containing 0.1% PVA, and permeabilized for 1 hour in PBS with 0.25% Triton X‐100 and 2% BSA. All antibodies were diluted in 1× PBS containing 2% BSA. Staining was performed overnight at 4 °C (in some conditions we used 2 hours at room temperature). Nuclei were stained by DAPI. Prolong Gold antifade mounting medium (Thermo Fisher Scientific, P36930) was used to mount embryos under the cover glass.

### Single‐Cell Collection and Single‐Cell RNA‐seq (scRNA‐seq)

Chimeras were collected and washed with 3 drops of cold DMEM/F12. Chimeras were transferred to enzymatic drops containing 6 units mL^−1^ of papain (Sigma‐Aldrich, P3125) in PBS containing 0.2 mg mL^−1^ cysteine, 5 mg mL^−1^ glucose, 0.4 mg mL^−1^ BSA and 0.5% Trypsin with ratio of 1:1 at 37 °C for 18 min. Gentle and repeated pipetting was performed, and embryos were incubated for another 10–15 min at 37 °C. Gentle and repeated pipetting was done again and embryos were ground gently. When all the cells in chimeras were observed to be partially dissociated, they were transferred to a 96‐well round‐bottom well for pipetting very gently 7–8 times. When single cells were almost observed, they were suspended in 2% BSA/1× PBS with 10 × 10^−6^ m Y‐27632 and washed by centrifugation at 500 × g for 6 min. The cells were resuspended in 0.1% BSA and placed on ice for the preparation of the single‐cell cDNA libraries (10x Genomics Genomics BioSci., & Tech.)

### Metabolite and Lipid Analysis

The metabolite and lipid standards were dissolved in cold MeOH at the final 50 ppm for LC‐MS/MS method establishment and qualification. The metabolite standards were Acetyl‐CoA (Sigma, A2056), HMG‐CoA (Sigma, H6132), Mevalonate (Sigma, M4667), Farnesyl pyrophosphate (FPP, Sigma, F6892). Hydrophilic compounds—Acetyl‐CoA, and HMG‐CoA have separated the HILIC Z column and measured in the positive mode of Orbitrap Elite Hybrid Mass Spectrometer. Hydrophilic compounds—Mevalonate, and FPP have separated the HILIC Z column and measured in the negative mode. The lipid standards—Cholesterol (Sigma, C8667), Desmosterol (Sigma, D6513), Lanosterol (Sigma, L5768), and Squalene (Sigma, S3626) were separated by the CSH‐C18 column and measured in the positive mode. Peaks detected by Orbitrap Elite‐MS were extracted using Thermo Xcalibur 4.1 software to obtain peak information, including *m*/*z*, retention time (RT), and peak area. Mass tolerance was set to 20 ppm and mass precision to 4 decimals. The tolerance window for the peak annotation was set to ± 15 s for RT and ± 5 min for view width. The Peak area of each metabolite was detected by the genesis method in the MS2 spectrum for further calculation. Samples in two biological repeats were analyzed in three technical repeats (hydrophobic, positive mode; hydrophilic, positive mode) and two technical repeats (hydrophilic, negative mode) and normalized to cell amounts. Metabolite qualification of each compound is as follows: Acetyl‐CoA ([M+H], *m*/*z* 810.1336; RT 6.09; MS2 *m*/*z* 428.0375@CID25), HMG‐CoA ([M+H], *m*/*z* 912.1653; RT 6.87; MS2 *m*/*z* 405.1664@CID25), Mevalonate ([M‐H], *m*/*z* 147.066; RT 3.80; MS2 *m*/*z* 59.0142@CID35), FPP ([M‐H], *m*/*z* 381.123; RT 4.49; MS2 *m*/*z* 363.114@CID35), Cholesterol ([M+H‐H2O], *m*/*z* 369.352165; RT 7.52; MS2 *m*/*z* 243.2109, 161.1325@CID30), Desmosterol ([M+H‐H2O], *m*/*z* 367.336465; RT 7.18; MS2 *m*/*z* 257.2268, 255.2111@CID30), Lanosterol ([M+H‐H2O], *m*/*z* 409.383465; RT 7.64; MS2 *m*/*z* 217.1951, 203.1792@CID30), Squalene ([M+H], *m*/*z* 411.399125; RT 8.75; MS2 *m*/*z* 231.2108, 217.1953@CID30).

### Lipid Raft Proteomics Mass Spectrometry and Data Processing

0.1% formic acid (FA) was used to suspend the samples. The samples were later loaded onto a C18‐Stage‐Tip column. Following the manufacturer's instructions, TMT 6‐Plex reagents (Thermo Fisher Scientific, 90061) were used to mark the samples. The lipid raft samples from GFP‐overexpressing cells, lipid raft samples from PODXL‐overexpressing cells, non‐raft membrane samples from GFP‐overexpressing cells, and nonraft membrane samples from PODXL‐overexpressing cells were labeled separately with TMT reagents 126, 127, 128 and 130, respectively, and the four samples were then pooled and concentrated in a SpeedVac. Then, peptides were separated into eight fractions with a High pH Reversed‐Phase Peptide Fractionation Kit (Thermo Fisher Scientific, 84868). Each fraction was spin‐dried in a Speed Vac and then frozen down to −80 °C until LC‐MS/MS analyses were performed in an LTQ‐Orbitrap Elite instrument (Thermo Fisher Scientific). MS2 spectra were searched against the UniProt *Homo sapiens* protein database using Proteome Discoverer (ver. 2.1, Thermo) and Mascot (ver. 2.5.01, Matrix Sciences) software. The Mascot settings included the following: 2 missed tryptic cleavages; assigned charges of +2 and +3; decoy database search (strict target FDR = 0.01, relaxed target FDR = 0.05); precursor and fragment mass tolerances of 10 ppm and 0.02 Da, respectively; and fixed modifications of carbamidomethylation of cysteines (C), oxidation of methionines (M), TMT6‐plex on peptide N‐terminal and TMT6‐plex on lysines (K). A basic reporter ion‐based quantification workflow was followed with Proteome Discoverer (ver 2.1, Thermo). No normalization and no scaling were performed. The fold changes were calculated from the relative protein abundance ratios. The threshold of the fold change ratios was set to greater than 2.00 for upregulation and less than 0.50 for downregulation. Proteins with a greater than fourfold change in the abundance ratio as identified by mass spectrometry analyses of the lipid raft fraction from PODXL‐overexpressing cells were subjected to PANTHER pathway analyses.

### Bulk RNA‐seq Data Preprocessing and Quality Control

RNA‐seq samples were pseudoaligned to the Ensembl GRCh38 human transcriptome release 90 using Kallisto, version 0.46.2.^[^ ^44^ ^]^ Transcript abundance was quantified with 100 bootstraps using default k‐mer length 31, mean fragment length 250, and sd 50. Transcripts per million (TPM) were log2(TPM + 1). We standardized and changed the row Z‐scores per gene for visualization.

### scRNA‐seq Data Preprocessing and Quality Control

Raw sequencing data were processed with Cell Ranger count (10x Genomics) using custom Ensembl Homo sapiens. GRCh38.104 and Mus_musculus.GRCm39.104 reference genomes appended with exogenous tdTomato and BSD_WPRE sequences. All data were first aligned to the custom mouse reference sequence and cells with no tdTomato and BSD_WPRE counts were defined as mouse cells. All data were separately aligned to the custom human reference sequence and cells with > 0 tdTomato and BSD_WPRE counts were defined as human cells.

### Clustering, Lineage Identification, and Pseudotime Trajectory Analyses

Seurat v3.2.1 was used to analyze all single‐cell data.^[^ ^45^ ^]^ Mouse cells with < 6000 unique genes detected and < 25% mitochondrial counts were retained for further analysis. Human cells with < 20% mitochondrial counts were retained for further analysis. Data were normalized and percent mitochondrial counts regressed out using the “SCTransform” function in Seurat. PCA and UMAP dimensionality reduction were performed using the first 30 empirically selected PCs with standard pipelines. Cluster markers were identified using the “FindAllMarkers” function in Seurat. Pseudotime trajectory analysis was performed using Monocle 3 v0.1.1,^[^ ^46^ ^]^ with standard preprocessing, dimensionality reduction, clustering, trajectory graph learning, and pseudotime ordering functions.

### Cell Type Proportion Analyses

The proportion of EPSC human cells within the chimeric embryos was quantified as the percentage of human cells recovered as a fraction of the total mouse and human cells recovered per −Dox and +Dox conditions. Similarly, the cell type proportions of EPSC mouse and human cells were quantified as the percentage of cells per cell type as a fraction of total mouse or human cells recovered per −Dox and +Dox conditions.

### Correlation Analyses

To compare transcriptomic signatures of EPSC mouse and human cells to primary mouse and human cells, Spearman correlation analyses per lineage were performed using the published mouse^[^ ^47^ ^]^ and human^[^ ^48^ ^]^ datasets. Normalized expression values for all single cells per lineage were averaged for each dataset and correlation analyses were performed by calculating Spearman correlation coefficients in the space of shared one‐to‐one mouse‐human gene orthologs across the whole transcriptome of all datasets.

### Identification of Inferred Cell–Cell Communication Interactions within Chimeras

The ligand‐receptor signaling cross‐talk between human and mouse cells in all lineages was built using CellChat (v1.1.3).^[^ ^26^ ^]^ All the ligand‐receptor interaction databases were included in CellChat analyses. In the function the “computeCommunProb,” we set population. size = TRUE to remove the bias of the proportion of cells in each group across all sequenced cells. In the function “filterCommunication,” the minimal cell numbers in each lineage were filtered out by fewer than 10 cells in chimeric mouse cells and accepted all the chimeric human cells. The other parameters were set as default. All the inferred cell–cell communications were used as the function 'subsetCommunication’ at the level of ligands/receptors.

### Statistical Analyses

For preprocessing of data, we performed gene expression normalization using GAPDH in qRT‐PCR data. Values were presented as the means ± SDs or means ± SEMs and the sample size of replicates (*n*) for each statistical analysis w also described in the figure legends. Comparison of means was performed using a two‐tailed, unpaired Student's t‐test for two group comparisons. One‐way ANOVA (three or more groups’ comparison) with Dunnett's Post hoc tests was done to compare all groups to the indicated control group. Testing levels using *p* < 0.05 was considered to indicate statistical significance (****p < 0.0001, ***p < 0.001, **p < 0.01, *p < 0.05). All figures were generated and statistical analyses were performed using GraphPad Prism 8.

## Conflict of Interest

Jean Lu and Wei‐Ju Chen have applied for a patent entitled, “METHODS FOR REGULATING POTENCY OF PLURIPOTENT STEM CELLS AND APPLICATIONS THEREOF.” The other authors declare no conflict of interest.

## Author Contributions

W.‐J.C., W.‐K.H., and S.R.P. contributed equally to this work. W.‐J.C., W.‐K.H., and J.L. conceived the idea for this project. W.‐J.C. conducted experiments, analyzed the data, and prepared the manuscript. S.R.P. performed the data analyses of RNA sequencing. W.‐F.C. and Li.‐Y.S. performed the embryo microinjection experiment. H.‐C.W., M.‐Y.L., C.‐C.L., and H.‐H.W. performed and analyzed the hESC monoclonal antibody experiment. C.‐Y.L., S.‐C.Y., H.L., P.‐L.L., C.‐H.N., F.L.L., and Y.‐C.L. conducted part of the experiments or provided reagents. C.‐M.H. and I.‐C.C. provided the NODSCID mouse. C.‐M.H, I.‐C.C., C.‐H.C., C.‐Y.L., and P.‐Y.L. provided reagents. A.S. provided iPSC reprogramming vectors. W.‐J.C., S.C.S., S.R.P., and J.L. wrote and edited the manuscript. Part of this paper was published in W.‐J.C. Ph.D. thesis.

## Code Availability

Key open‐access versions of the programs and pipeline used in this paper are available by GitHub at Seurat^[^ ^45^ ^]^ (https://www.github.com/satijalab/seurat), Monocle3^[^ ^46^ ^]^ (https://www.github.com/cole‐trapnell‐lab/monocle3), and CellChat^[^ ^26^ ^]^ (https://www.github.com/sqjin/CellChat).

## Supporting information

Supporting Information

## Data Availability

The microarray data used in this paper is GSE127218. All raw and processed scRNA‐seq data are available from the GEO database under accession number GSE189014. Furthermore, published datasets were downloaded as provided by Wen et al.^[^ ^47^ ^]^ (GEO: GSE70713) and Zhou et al.^[^ ^48^ ^]^ (GEO: GSE109555). Bulk sequence data was available at GSE213618.
