# Supplementary material for: Podocalyxin‐Like Protein 1 Regulates Pluripotency through the Cholesterol Biosynthesis Pathway
Source: Adv Sci (Weinh). 2022 Nov 14;10(1):2205451. doi: 10.1002/advs.202205451 (PMC9811443; doi:10.1002/advs.202205451)
Supplement: Supplementary file 1 — Supporting Information [file ADVS-10-2205451-s001.pdf]

Supporting Information

Podocalyxin-Like Protein 1 Regulates Pluripotency through the Cholesterol Biosynthesis Pathway

Wei-Ju Chen, Wei-Kai Huang, Sarshan R. Pather, Wei-Fang Chang, Li-Ying Sung, Han-Chung Wu, Mei-Ying Liao, Chi-Chiu Lee, Hsuan-Hui Wu, Chung-Yi Wu, Kuo-Shiang Liao, Chun-Yu Lin, Shang-Chih Yang, Hsuan Lin, Pei-Lun Lai, Chi-Hou Ng, Chun-Mei Hu, I-Chih Chen, Chi-Hsuan Chuang, Chien-Ying Lai, Po-Yu Lin, Yueh-Chang Lee, Scott C. Schuyler, Axel Schambach, Frank Leigh Lu and Jean Lu*

*Corresponding author

W.-J.Chen, J.Lu
Genomics Research Center
Academia Sinica
Genome and Systems Biology Degree Program
College of Life Science
National Taiwan University
Taipei 10617, Taiwan
W.-K. Huang
Center for Genomic Medicine
Massachusetts General Hospital
Boston, MA 02114, USA
S.R. Pather
Cell and Molecular Biology Graduate Group
Perelman School of Medicine
University of Pennsylvania
Philadelphia, PA 19104, USA

E-mail:jeanlu@gate.sinica.edu.tw

W.-J. Chen, K.-S. Liao, C.-Y. H. Lin, S.-C Yang, P.-L. Lai, C.-H. Ng, C.-M. Hu, I.-C. Chen, C.-H. Chuang, C.-Y.Lai, P.-Y.Lin, J. Lu
Genomics Research Center
Academia Sinica
Taipei 11529, Taiwan
W.-F Chang, L.-Y Sung
Institute of Biotechnology
National Taiwan University
Taipei 10617, Taiwan
L.-Y Sung
Agricultural Biotechnology Research Center
Academia Sinica
Taipei 11529, Taiwan
Animal Resource Center
National Taiwan University
Taipei 10617, Taiwan
H.-C Wu, M.-Y Liao, C.-C Lee, H.-H Wu
Institute of Cellular and Organismic Biology

Academia Sinica
Taipei 11529, Taiwan
H.-C Wu
Biomedical Translation Research Center (BioTReC)
Academia Sinica
Taipei, 11529, Taiwan
Y.-C. Lee
Department of Ophthalmology
Hualien Tzu Chi Hospital
Buddhist Tzu Chi Medical Foundation
Hualien 97004, Taiwan
S.C. Schuyler
Department of Biomedical Sciences
College of Medicine
Chang Gung University
Division of Head and Neck Surgery
Department of Otolaryngology
Chang Gung Memorial Hospital
Taoyuan 33302, Taiwan
A. Schambach
Institute of Experimental Hematology
Hannover Medical School
Hannover 30625, Germany
F.L. Lu
Department of Pediatrics
National Taiwan University Hospital and National Taiwan University Medical College
Taipei 10051, Taiwan
J. Lu
National RNAi Platform/ National Core Facility Program for Biotechnology
Taipei 11529, Taiwan
Department of Life Science
Tzu Chi University
Hualien 97004, Taiwan
Graduate Institute of Medical Sciences
National Defense Medical Center
Taipei 11490, Taiwan

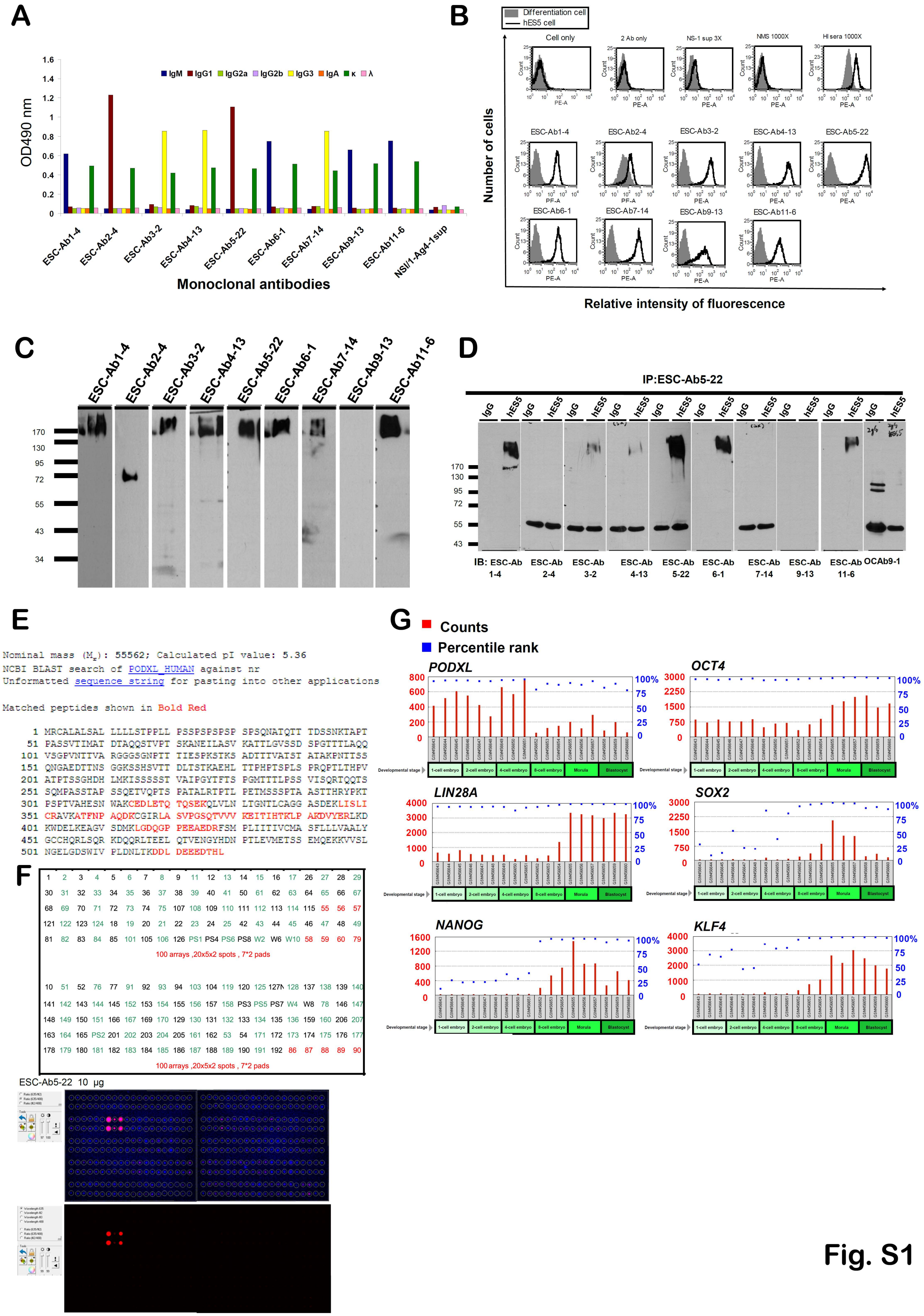
A
IgM IgG1 IgG2a IgG2b IgG3 IgA κ λ
OD490 nm
ESC-Ab1-4
ESC-Ab2-4
ESC-Ab3-2
ESC-Ab4-13
ESC-Ab5-22
ESC-Ab6-1
ESC-Ab7-14
ESC-Ab9-13
ESC-Ab11-6
NSI/1-Ag4-1sup
Monoclonal antibodies
B
Differentiation cell
hES5 cell
Cell only
2 Ab only
NS-1 sup 3X
NMS 1000X
HI sera 1000X
Number of cells
Relative intensity of fluorescence
C
D
IP:ESC-Ab5-22
IB: ESC-Ab 1-4
ESC-Ab 2-4
ESC-Ab 3-2
ESC-Ab 4-13
ESC-Ab 5-22
ESC-Ab 6-1
ESC-Ab 7-14
ESC-Ab 9-13
ESC-Ab 11-6
OCAb9-1
E
Nominal mass (Mr): 55562; Calculated pI value: 5.36
NCBI BLAST search of PODXL_HUMAN against nr
Unformatted sequence string for pasting into other applications
Matched peptides shown in Bold Red
1 MRCALALSAL LLLLSTPPLL PSSPSPSPSP SPSQNATQTT TDSSNKTAPT
51 PASSVTIMAT DTAQQSTVPT SKANEILASV KATTLGVSSD SPGTTTLAQQ
101 VSGPVNTTVA RGGGSGNPTT TIESPKSTKS ADTTTVATST ATAKPNTTSS
151 QNGAEDTTNS GGKSSHSVTT DLTSTKAEHL TTPHPTSPLS PRQPTLTHPV
201 ATPTSSGHDH LMKISSSSST VAIPGYTFTS PGMTTTLPSS VISQRTQQTS
251 SQMPASSTAP SSQETVQPTS PATALRTPTL PETMSSSPTA ASTTHRYPKT
301 PSPTVAHESN WAKCEDLETQ TQSEKQLVLN LTGNTLCAGG ASDEKLISLI
351 CRAVKATFNP AQDKCGIRLA SVPGSQTVVV KEITIHTKLP AKDVYERLKD
401 KWDELKEAGV SDMKLGDQGP PEEAEDRFSM PLIITIVCMA SFLLLVAALY
451 GCCHQRLSQR KDQQRLTEEL QTVENGYHDN PTLEVMETSS EMQEKKVVSL
501 NGELGDSWIV PLDNLTKDDL DEEEDTHL
F
100 arrays ,20x5x2 spots , 7*2 pads
ESC-Ab5-22 10 μg
G
Counts
Percentile rank
PODXL
OCT4
LIN28A
SOX2
NANOG
KLF4
Developmental stage
1-cell embryo
2-cell embryo
4-cell embryo
8-cell embryo
Morula
Blastocyst

Fig. S1

Figure S1. Characterization of mAbs and the targeted proteins related to Figure 1.
(A) Isotype determination of mAbs. ELISA tests showed that ESC-Ab-1-4, ESC-Ab-6-1, ESC-Ab-9-13 and ESC-Ab-11-6 belong to the IgM subclass. ESC-Ab-2-4 and ESC-Ab-5-22 belong to the IgG1 subclass. ESC-Ab-3-2, ESC-Ab-4-13, and ESC-Ab-7-14 belong to the IgG3 subclass.
(B) Flow cytometric analyses of mAbs binding to HUES5 cells.
(C) mAbs ESC-Ab-1-4, -2-4, -3-2, -4-13, -5-22, -6-1, -7-14 and -11-6 recognized HUES5 cells using a reducing gel.
(D) Western blot analyses showed immuno-complexes co-precipitated by ESC-Ab-5-22 mAb. OCAb-9-1 was against EpCAM.
(E) LC-MS/MS protein identification of the ESC-Ab-5-22 target protein as PODXL.
(F) Total glycan array print formats and fluorescence images were shown. Spots 36 and 38 have positive signals.
(G) *PODXL* expression levels were abundant from the one-cell embryo to the 4-cell embryo stages. The expression levels of the pluripotency genes *OCT4*, *SOX2*, *NANOG*, *KLF4,* and *LIN28A* were highest in the morula and blastocyst stages. Data were derived from the Gene Expression Omnibus (GEO) dataset GSE18290.

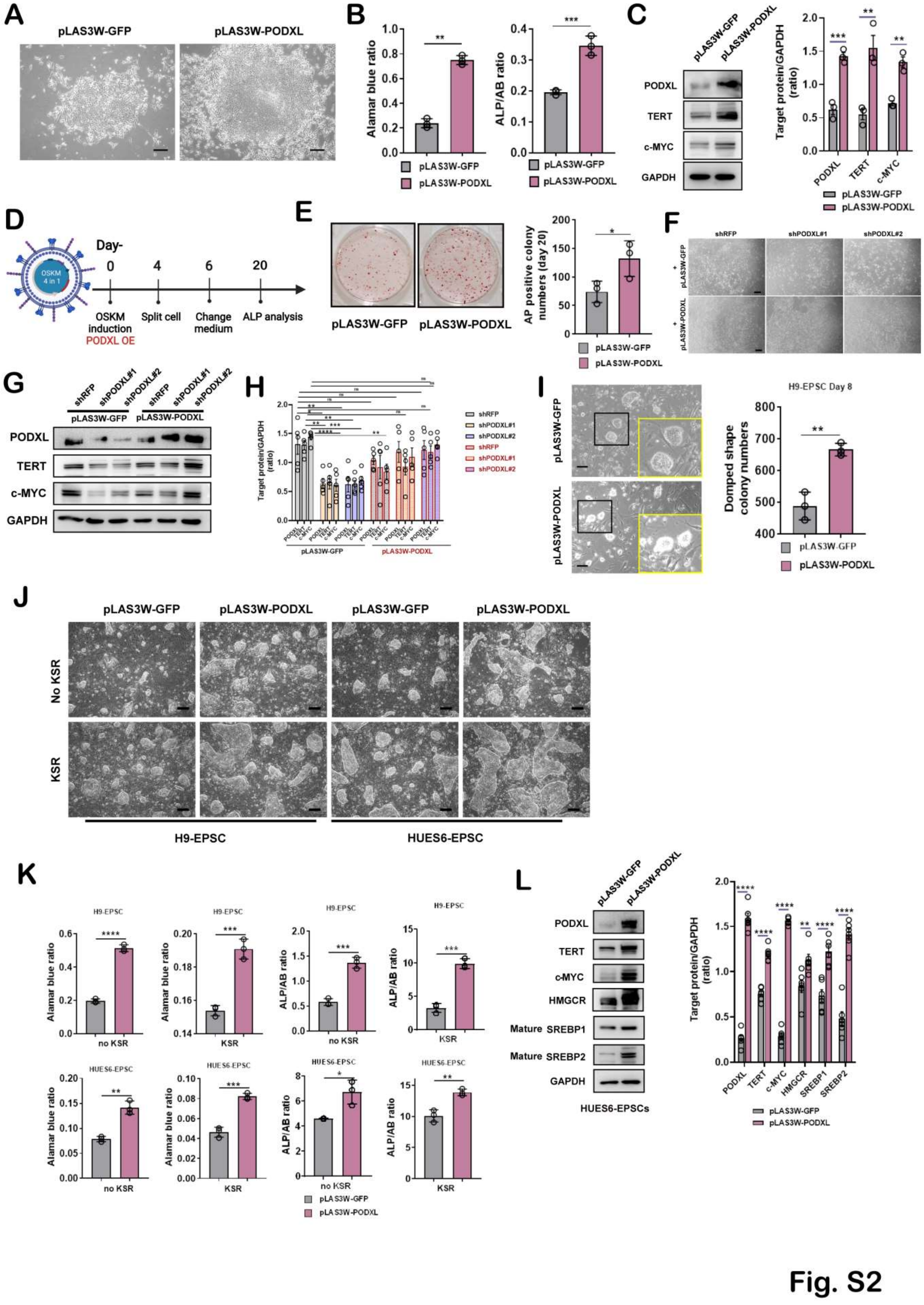

Figure S2. PODXL overexpression promotes self-renewal of hESCs and hEPSCs, and iPSC generation, related to Figure 2.
(A) Bright-field images of ectopic PODXL expression in HUES6 hESCs and GFP control hESCs.

(B) AB assays and ALP activity normalized to relative cell numbers (AB assays) performed on PODXL-overexpressing HUES6-hESCs and control GFP hESCs. Unpaired Student's t-test (***p < 0.001, **p < 0.01) was performed relative to pLAS3W-GFP control hESCs.
(C) Quantification of the Western blots showing PODXL/TERT/c-MYC. The error bars indicate the averages ± SEMs (n = 3). Unpaired Student's t-test (***p < 0.001, **p < 0.01) was performed relative to pLAS3W-GFP control HUES6 hESCs.
(D) Flow chart of iPSC generation.
(E) Reprogrammed colonies were stained to assess ALP, and ALP-positive colonies were counted. The error bars indicate the averages ± SDs (n = 3). Unpaired Student's t-test (*p < 0.05) was performed relative to pLAS3W-GFP control group.
(F) Bright-field images of the PODXL knockdown phenotype (HUES6 cells) rescued by co-infection with pLAS3W-PODXL lentivirus. hESCs infected with pLAS3W-PODXL or pLAS3W-GFP lentivirus for 7 days. Scale bar, 200 µm.
(G-H) Western blots showing the rescue of c-MYC and TERT expression after forced PODXL expression in PODXL knockdown HUES6 hESCs. The error bars indicate the averages ± SEMs (n = 5). P-values were determined by one-way ANOVA (****p < 0.0001, ***p < 0.001, **p < 0.01, *p < 0.05, n.s.: not significant) relative to shRFP-pLAS3W-GFP control hESCs.
(I) PODXL overexpression increases the number of H9 EPSCs with a domed shape. Bright-field images of PODXL-overexpressing hEPSCs and GFP control hEPSCs on day 8 of extended pluripotency conversion. Quantification of dome-shaped colony numbers of control or PODXL-overexpressing hESCs on day 8 of culture under extended pluripotency conditions (N2B27-LCDM). The error bars indicated the averages ± SDs (n = 3). Unpaired Student's t-test (**p < 0.01) was performed relative to pLAS3W-GFP control hEPSCs.
(J) Bright-field images of PODXL-overexpressing H9 and HUES6-hEPSCs on day 9. KSR: 5% KnockOut serum replacement.
(K) PODXL overexpression increased the relative cell numbers of EPSCs (AB assays) and the expression of stem cell markers (ALP/AB activity). Basal medium containing 5% KSR had a smaller OD value in the AB assay compared to the no KSR condition, therefore the AB ratio cannot be compared directly between no KSR and KSR conditions. The error bars indicate the averages ± SDs (n = 3). Unpaired Student's t-test (**** < 0.0001, ***p < 0.001, **p < 0.01, *p < 0.05) was performed relative to pLAS3W-GFP control hEPSCs.
(L) Overexpression of PODXL increased c-MYC, TERT, HMGCR, SREBP1, and SREBP2 expression in HUES6-EPSCs. The error bars indicate the averages ± SEMs (n = 3, for both H9-EPSCs and HUES6-EPSCs). P-values were determined with an unpaired Student's t-test (****p < 0.0001, ***p < 0.001, **p < 0.01) was performed relative to GFP control hEPSCs.

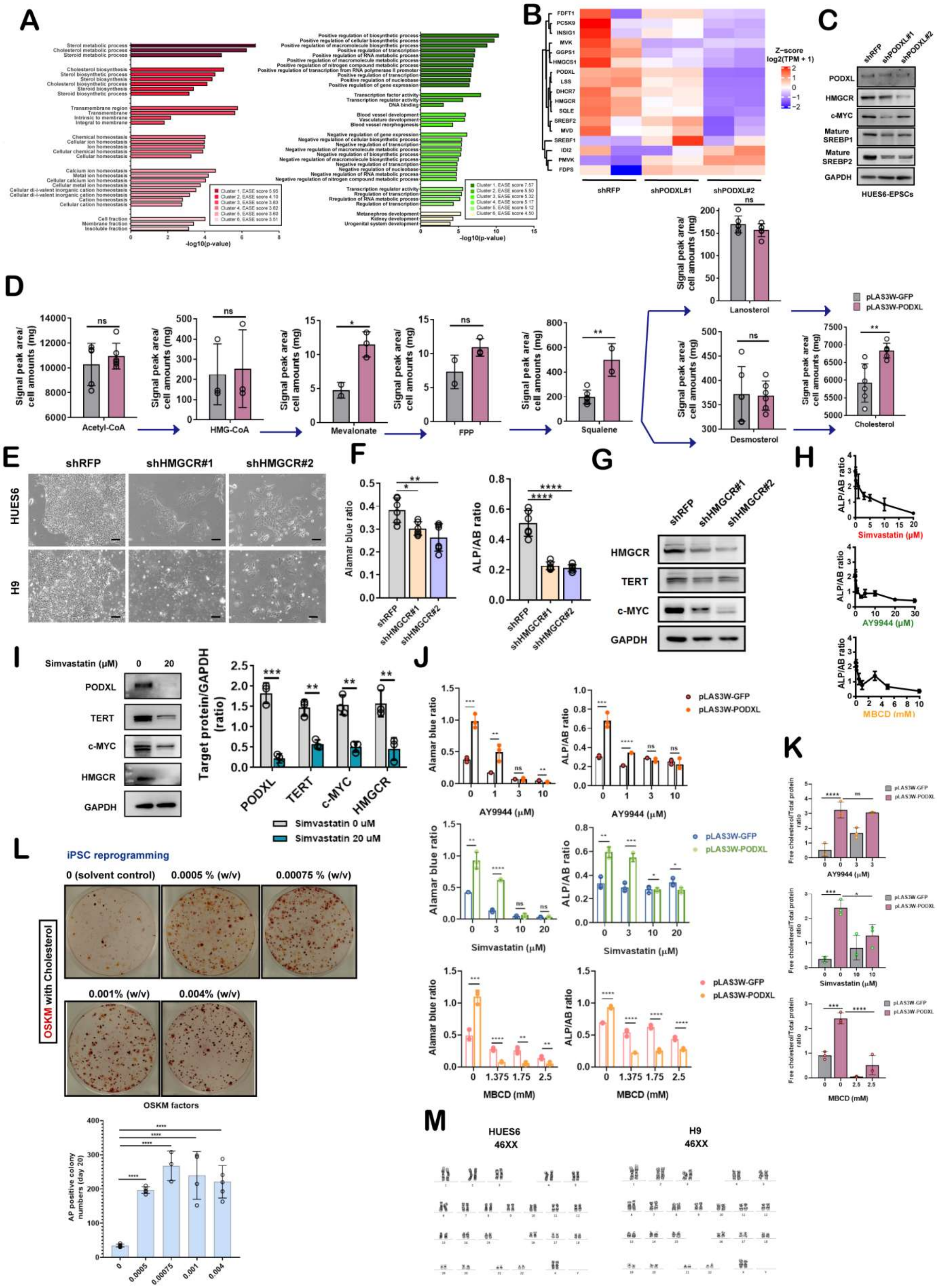

Fig. S3

Figure S3. PODXL regulates the cholesterol pathway through HMGCR and cholesterol is pivotal for hPSC self-renewal, related to Figure 3.

(A) GO terms enriched in genes upregulated and downregulated in PODXL-overexpressing

HUES6 cells. GO functional annotation of genes upregulated or downregulated by 1.5-fold was performed using the DAVID functional annotation tool. The EASE score, a modified Fisher's exact p-value, is shown ($p < 0.001$).
(B) Heatmap representation of shPODXL RNA-Seq in HUES6. Z-score on TPM-level data was computed for gene expressions which were involved in regulation of cholesterol biosynthetic process. Two biological replicates were conducted in each condition.
(C) Western blots showing downregulation of c-MYC, HMGCR, SREBP1 and SREBP2 expression in HUES6-derived EPSCs with PODXL knockdown after 7 days of shRNA lentiviral transduction.
(D) Relative quantification of cholesterol metabolites by LC-MS/MS analysis in PODXL-overexpressing HUES6 hESCs. Two independent biological replicates were performed and analyzed by technical triplicates or duplicates at the same time. The error bars indicate the averages ± SDs. P-values were determined with an unpaired Student's t-test (**$p < 0.01$, *$p < 0.05$) relative to GFP control cells.
(E) Bright-field images of shHMGCR-transduced HUES6 and H9 hESCs.
(F) Downregulation of HMGCR reduced the relative cell numbers (Alamar blue assay) and the expression of pluripotency markers (ALP) in HUES6 hESCs. Assays were performed in shRNA-treated hESCs after 6 days of lentiviral transduction. P-values were determined with one-way ANOVA (****$p < 0.0001$, ***$p < 0.001$, **$p < 0.01$) with Dunnett's multiple comparisons test performed relative to shRFP hESCs.
(G) Western blots showing c-MYC and TERT expression in shHMGCR-transduced hESCs.
(H) ALP activity of HUES6 hESCs treated separately with simvastatin, AY9944 and MBCD for 3 days. Scale bar, 200 µm.
(I) Quantification of the Western blots showed that simvastatin blocks the expression of PODXL, TERT, c-MYC, and HMGCR. The error bars indicate the averages ± SEMs (n = 3). P-values were determined with an unpaired Student's t-test (***$p < 0.001$, **$p < 0.01$) relative to solvent control cells.
(J) Relative cell numbers (AB assays) and ALP activity (ALP/AB) were evaluated under treatment with increasing concentrations of the three inhibitors for 3 days upon PODXL overexpression of HUES6 cells. P-values were determined with an unpaired Student's t-test (****$p < 0.0001$, ***$p < 0.001$, **$p < 0.01$, *$p < 0.05$) relative to GFP control cells.
(K) The free cholesterol levels in HUES6 hESCs treated with three cholesterol inhibitors with PODXL overexpression, respectively. The error bars indicate the averages ± SDs (n = 3). P-values were determined with One-way ANOVA and Sidak's multiple comparisons test (****$p < 0.0001$, ***$p < 0.001$, **$p < 0.01$) was performed relative to solvent control cells (0) expressing GFP or PODXL.
(L) Cholesterol supplementation during iPSC generation. ALP staining was performed, and the number of AP-positive colonies was counted on day 20 post-OSKM lentiviral transduction. Free cholesterol was supplemented during iPSC reprogramming. Error bars indicate the averages ± SDs (n = 5 at concentration 0, n = 5 at concentration 1×10^{-4}, n = 3 at concentration 7.5×10^{-4}, n = 4 at concentration 1×10^{-3}, n = 5 at concentration is 4×10^{-3}). P-values were determined with one-way ANOVA (***$p < 0.001$, **$p < 0.01$, *$p < 0.05$) with Dunnett's multiple comparison test.
(M) Karyotype analysis of HUES6 and H9 cells.

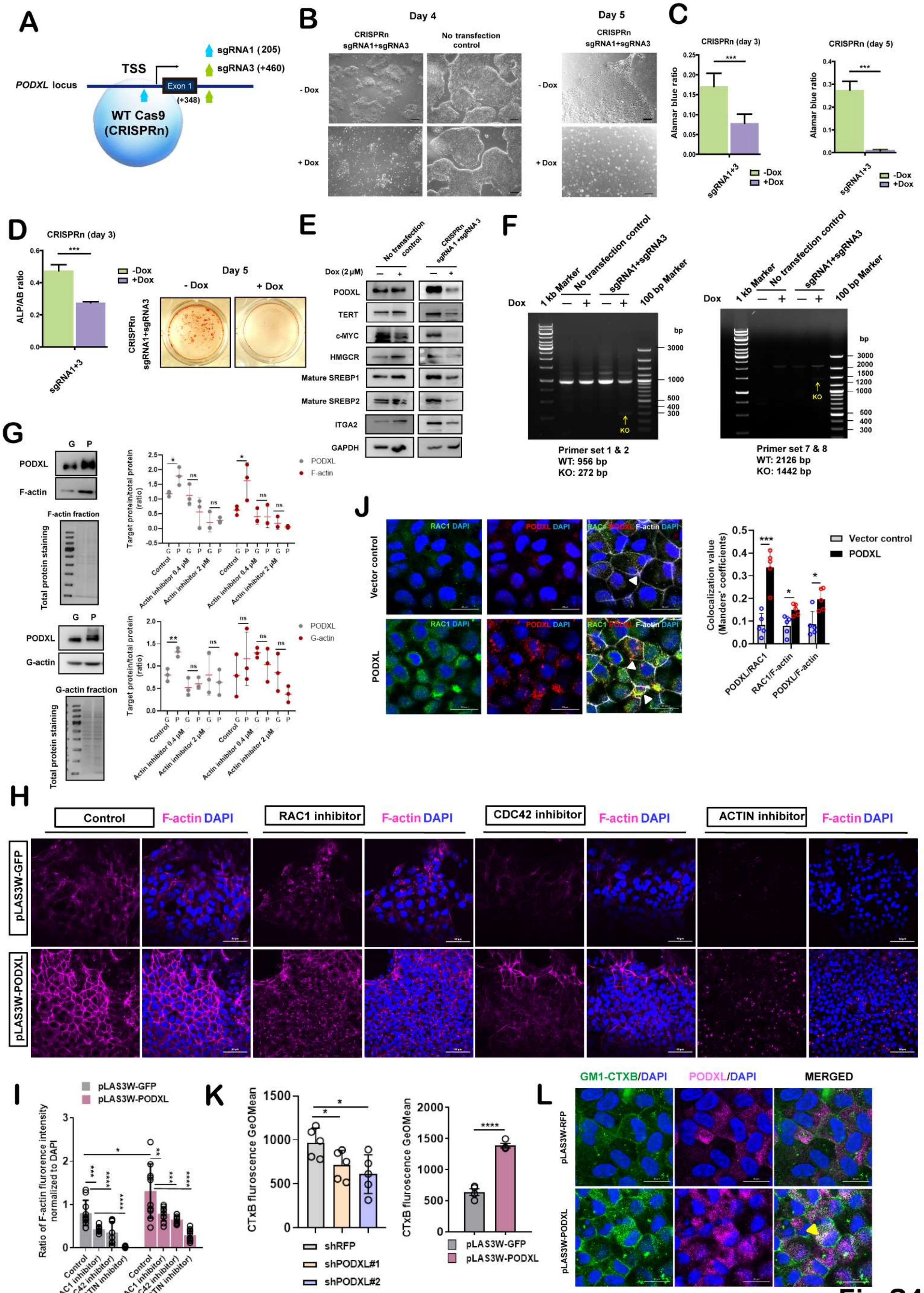
A
PODXL locus
TSS
Exon 1
(+348)
sgRNA1 (205)
sgRNA3 (+460)
WT Cas9 (CRISPRn)
B
Day 4
Day 5
CRISPRn sgRNA1+sgRNA3
No transfection control
- Dox
+ Dox
C
CRISPRn (day 3)
CRISPRn (day 5)
Alamar blue ratio
sgRNA1+3
-Dox
+Dox
D
CRISPRn (day 3)
ALP/AB ratio
Day 5
CRISPRn sgRNA1+sgRNA3
E
No transfection control
CRISPRn sgRNA 1 +sgRNA 3
Dox (2 μM)
PODXL
TERT
c-MYC
HMGCR
Mature SREBP1
Mature SREBP2
ITGA2
GAPDH
F
1 kb Marker
No transfection control
sgRNA1+sgRNA3
100 bp Marker
Dox
bp
KO
Primer set 1 & 2
WT: 956 bp
KO: 272 bp
Primer set 7 & 8
WT: 2126 bp
KO: 1442 bp
G
PODXL
F-actin
F-actin fraction
Total protein staining
G-actin
G-actin fraction
Target protein/total protein (ratio)
Control
Actin inhibitor 0.4 μM
Actin inhibitor 2 μM
J
RAC1 DAPI
PODXL DAPI
RAC1 PODXL F-actin DAPI
Vector control
PODXL
Colocalization value (Manders' coefficients)
PODXL/RAC1
RAC1/F-actin
PODXL/F-actin
H
Control
RAC1 inhibitor
CDC42 inhibitor
ACTIN inhibitor
F-actin DAPI
pLAS3W-GFP
pLAS3W-PODXL
I
Ratio of F-actin flurorence intensity normalized to DAPI
K
CTxB fluroscence GeOMean
shRFP
shPODXL#1
shPODXL#2
L
GM1-CTXB/DAPI
PODXL/DAPI
MERGED
pLAS3W-RFP
pLAS3W-PODXL

Fig.S4

Figure S4. PODXL regulates lipid raft dynamics through actin polymerization and cholesterol, related to Figure 4.

(A) Schematic overview of the generation of PODXL knockout hiPSC cell lines. sgRNAs were targeted to the 5'UTR and intron 1 sequences at the PODXL locus. A 537-bp of genomic fragment including exon 1 was deleted from the PODXL locus. The vertical arrows indicate the sgRNA1 and sgRNA3 target sites.

(B) Bright-field images of inducible PODXL knockout cells after drug selection for 4 days or 5 days, respectively.

(C) Relative numbers of cells with inducible PODXL knockout iPSCs after drug selection for 3 or 5 days. P-values were determined with an unpaired Student's t-test (***p < 0.001) relative to GFP control cells.

(D) ALP activity of cells with inducible PODXL knockout after drug selection for 3 days or ALP staining at day 5. P-values were determined with an unpaired Student's t-test (***p < 0.001) relative to GFP control cells.

(E) TERT, c-MYC, HMGCR, SREBP1, SREBP2, ITGA2 expression was downregulated upon PODXL knockout. Western blot validation of inducible PODXL knockout after drug selection for 4 days.

(F) Genomic deletion analyses in iPSCs with inducible PODXL knockout after drug selection for 5 days. The deleted form of the PCR product was indicated by a yellow arrow. WT: wild-type; KO: knockout.

(G) Overexpression of PODXL increased actin polymerization. F-actin and G-actin *in vivo* assays were performed after forced PODXL expression in HUES6 hESCs. The error bars indicate the averages ± SDs (n = 3). P-values were determined with an unpaired Student's t-test (**p < 0.01, *p < 0.05) relative to GFP control cells.

(H-I) Inhibition of RAC1 inhibitor (EHop-016), CDC42 inhibitor (ZCL278), actin inhibitor (Latrunculin A) reduced actin polymerization in GFP-control and PODXL-overexpressing HUES6 hESCs. Fluorescent phalloidins (Alexa Fluor-647 Phalloidin) were used for staining actin filament (F-actin) and analyzed by confocal microscopy. The error bars indicate the averages ± SDs (n = 10). An unpaired Student's unpaired t-test (****p < 0.0001, ***p < 0.001, **p < 0.01, *p<0.05) was performed relative to GFP control hESCs.

(J) The expression patterns of RAC1, PODXL, and F-actin in HUES6 hESCs. Representative confocal images of RAC1 (green), PODXL (red), and F-actin (white) are indicated as shown. Scale bars, 20 μm. Quantification of colocalization. Manders' coefficients represent the correlation between the cellular locations of RAC1 and PODXL, RAC1 and F-actin, and PODXL and F-actin. The white triangles indicate colocalized signals of RAC1, PODXL, and F-actin. The error bars indicate the averages ± SDs (n = 5). P-values were determined with an unpaired Student's t-test (***p < 0.001, *p < 0.05) Plugin tools JACoP in Image J was used to perform image quantification.

(K) Lipid raft abundance assessed in HUES6 hESCs with PODXL knockdown or PODXL overexpression was detected by CTxB-Alexa488 and quantified by flow cytometry. The error bars indicate the averages ± SEMs (n = 5 in shPODXL; n = 4 in PODXL overexpression). P-values were determined by one-way ANOVA (*p < 0.05) relative to shRFP control hESCs. An unpaired Student's unpaired t-test (****p < 0.0001) was performed relative to GFP control hESCs.

(L) Representative confocal images in HUES6 hESCs. Lipid rafts, CTxB-Alexa488 (GM1-CTxB, green) co-localized with anti-PODXL antibody (pink) staining indicating the PODXL membrane location relative to lipid rafts. Scale bars, 20 μm. The yellow triangle indicates a colocalized signal of PODXL and lipid rafts.

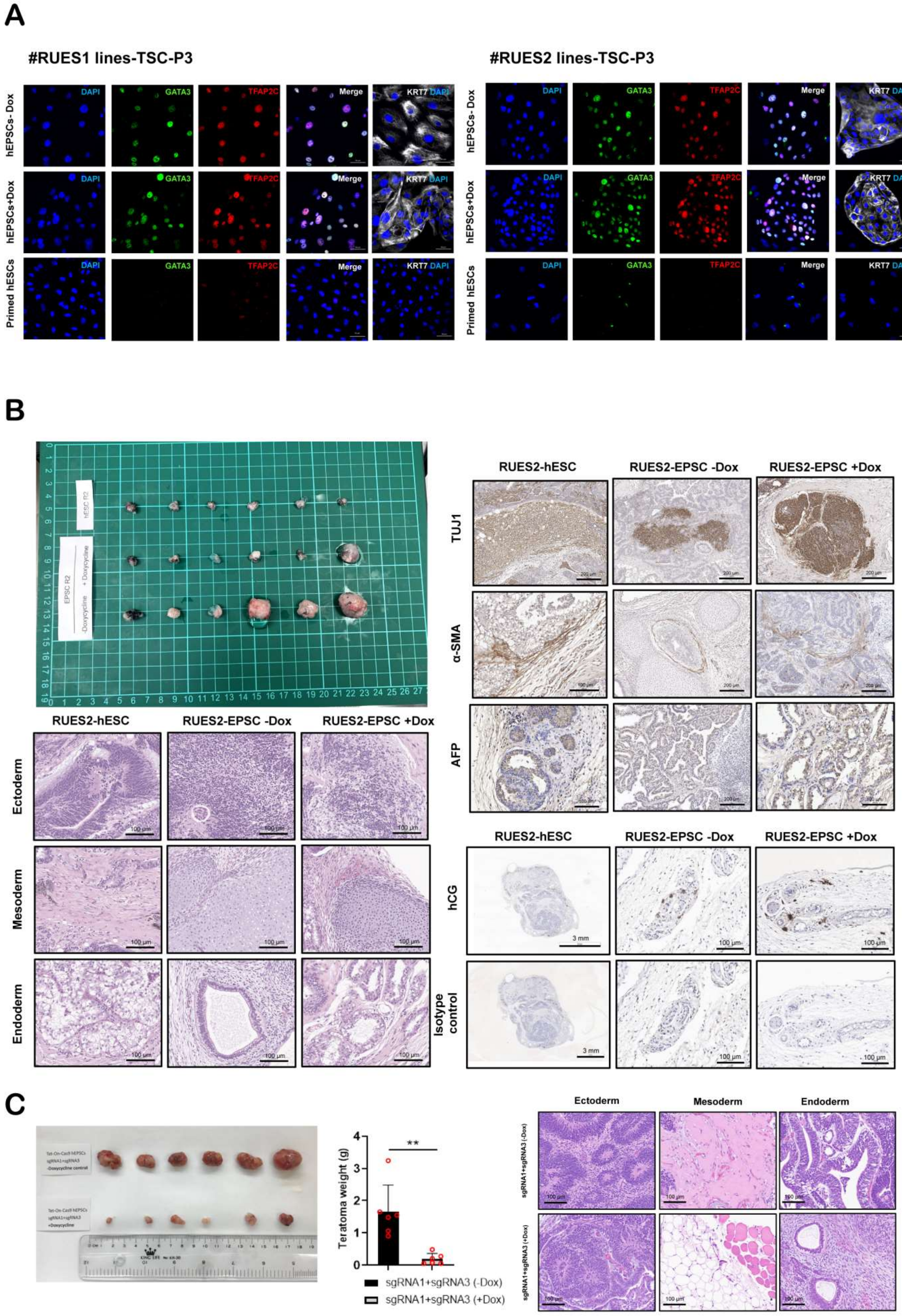
A
#RUES1 lines-TSC-P3
#RUES2 lines-TSC-P3
hEPSCs- Dox
hEPSCs+Dox
Primed hESCs
DAPI
GATA3
TFAP2C
Merge
KRT7 DAPI
B
RUES2-hESC
RUES2-EPSC -Dox
RUES2-EPSC +Dox
Ectoderm
Mesoderm
Endoderm
TUJ1
α-SMA
AFP
hCG
Isotype control
100 μm
200 μm
3 mm
C
Teratoma weight (g)
**
sgRNA1+sgRNA3 (-Dox)
sgRNA1+sgRNA3 (+Dox)
Ectoderm
Mesoderm
Endoderm

Fig.S5

Figure S5. *In vitro* TSC differentiation and *in vivo* teratoma formation of PODXL overexpression in the EPSC state, related to Figure 5.

(A) Immunofluorescence staining for TSC markers GATA3, TFAP2C, and KRT7 in hTSC-like cells derived from RUES1- and RUES2-hEPSCs or hESCs. The scale bars indicate 50 μm.

(B) Teratomas generated from engrafted cells (RUES2-hESCs, RUES2-EPSC -Dox, RUES2-EPSC +Dox) by subcutaneous injection. H&E staining and immunohistochemistry analyses for ectoderm, mesoderm, endoderm, and trophectoderm layers in teratomas were shown as indicated.

(C) Teratoma formation in inducible PODXL knockout in the EPSC state. Teratomas generated from engrafted cells (Tet-On-Cas9-EPSC -Dox sgRNA1+sgRNA3, Tet-On-Cas9-EPSC +Dox sgRNA1+sgRNA3) by subcutaneous injection. Dox, Doxycycline. Quantification of the weights of teratomas. The error bars indicate the averages ± SDs ($n = 6$). P-values were determined with an unpaired Student's t-test (**$p < 0.01$) relative to -Dox sgRNA1+sgRNA3 control group. H&E stainings for ectoderm, mesoderm, and endoderm in teratomas are shown as indicated.

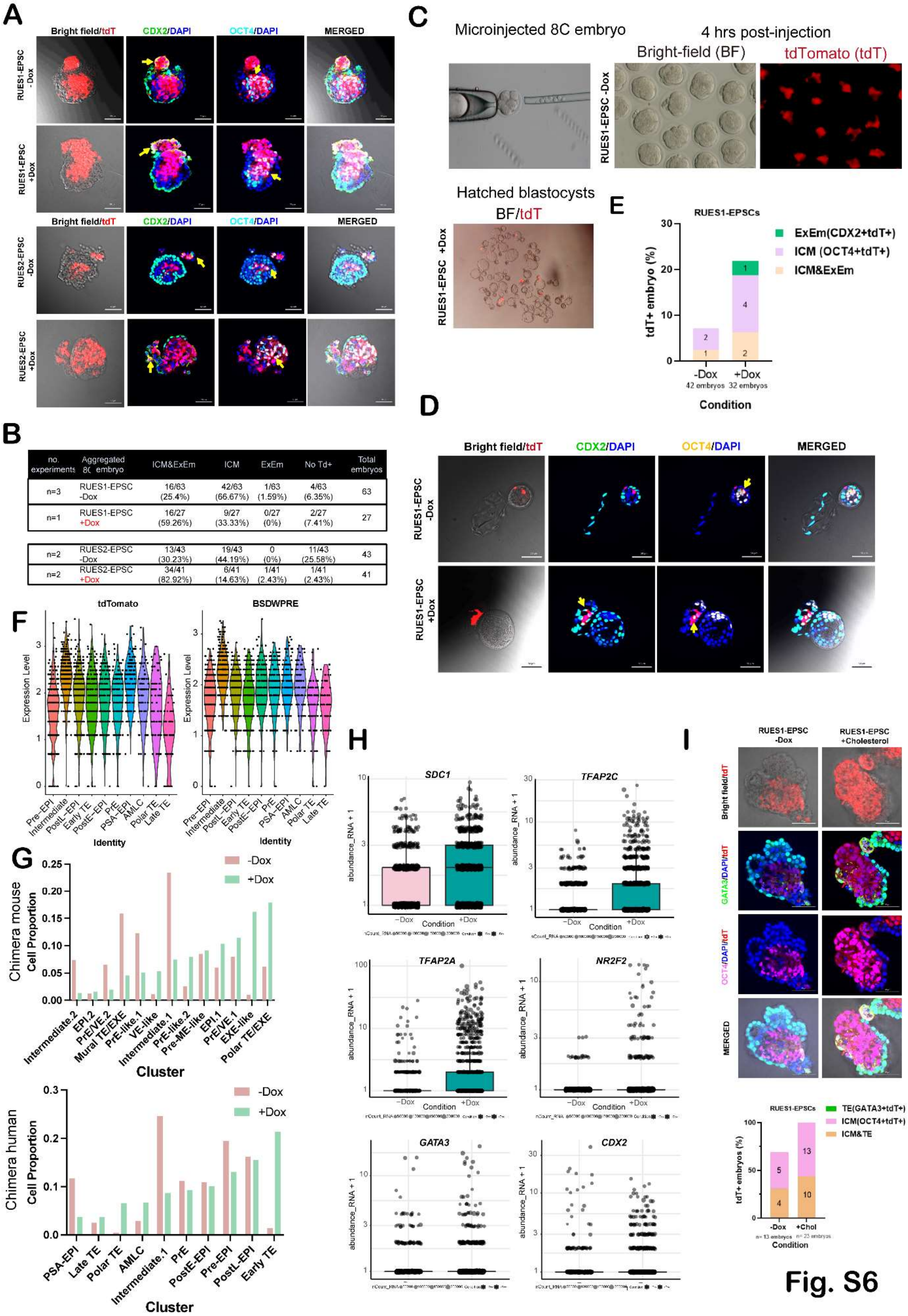

B

no. experiments	Aggregated 8C embryo	ICM&ExEm	ICM	ExEm	No Td+	Total embryos
n=3	RUES1-EPSC -Dox	16/63 (25.4%)	42/63 (66.67%)	1/63 (1.59%)	4/63 (6.35%)	63
n=1	RUES1-EPSC +Dox	16/27 (59.26%)	9/27 (33.33%)	0/27 (0%)	2/27 (7.41%)	27
n=2	RUES2-EPSC -Dox	13/43 (30.23%)	19/43 (44.19%)	0 (0%)	11/43 (25.58%)	43
n=2	RUES2-EPSC +Dox	34/41 (82.92%)	6/41 (14.63%)	1/41 (2.43%)	1/41 (2.43%)	41

Fig. S6

Figure S6. Inducing PODXL overexpression enhances human-mouse chimerism in peri-implantation stage embryos.

(A-B) Immunofluorescence staining, identification, and quantification of pre-implantation chimeric embryos using aggregation methods. The percentage of E4.5-E5.0 human-mouse chimeric embryos with different lineage contributions (ICM, ExEm, ICM&ExEm), stained for OCT4 (ICM) or CDX2 (ExEm). tTd, tdTomato. ExEm, extraembryonic tissue. RUES1- and RUES2-EPSCs were used in this assay.

(C) Representative view of embryo microinjection of RUES1 cells, 4 hours post-injection and further development for ~24-37 hours.

(D-E) Immunofluorescence staining identification and quantification of pre-implantation chimeric embryos using microinjection method. The percentage of E4.5-E5.0 human-mouse chimeric embryos with different lineage contributions (ICM, ExEm, ICM, and ExEm), stained for OCT4 (ICM), CDX2 (ExEm). tTd, tdTomato. ExEm, extraembryonic tissue. RUES1-EPSCs were used in this assay.

(F) The expression levels of introduced tdTomato and BSDWPRE in each cell cluster in chimeric human RUES1-EPSCs.

(G) Bar plot of cell proportions in each cell cluster in chimeric human and chimeric mouse embryos for each different experimental condition (-Dox or +Dox). RUES1-EPSCs were used in this assay.

(H) Box and whiskers plot of RNA abundance of TE marker genes for each condition (-Dox or +Dox). The upper whisker extends from the hinge to the highest value that is within 1.5 * IQR of the hinge, where IQR is the inter-quartile range or distance between the first and third quartiles. The lower whisker extends from the hinge to the lowest value within 1.5 * IQR of the hinge. Each dot represents every single cell. RUES1 cells were used in this assay.

(I) Effect of cholesterol supplementation in human-mouse chimeras. Immunofluorescence staining identification and quantification of pre-implantation chimeric embryos using the aggregated method. The percentage of E4.5-E5.0 human-mouse chimeric embryos with different lineage contributions (ICM, TE, ICM and TE), stained for OCT4 (ICM), GATA3 (TE). tTd, tdTomato.

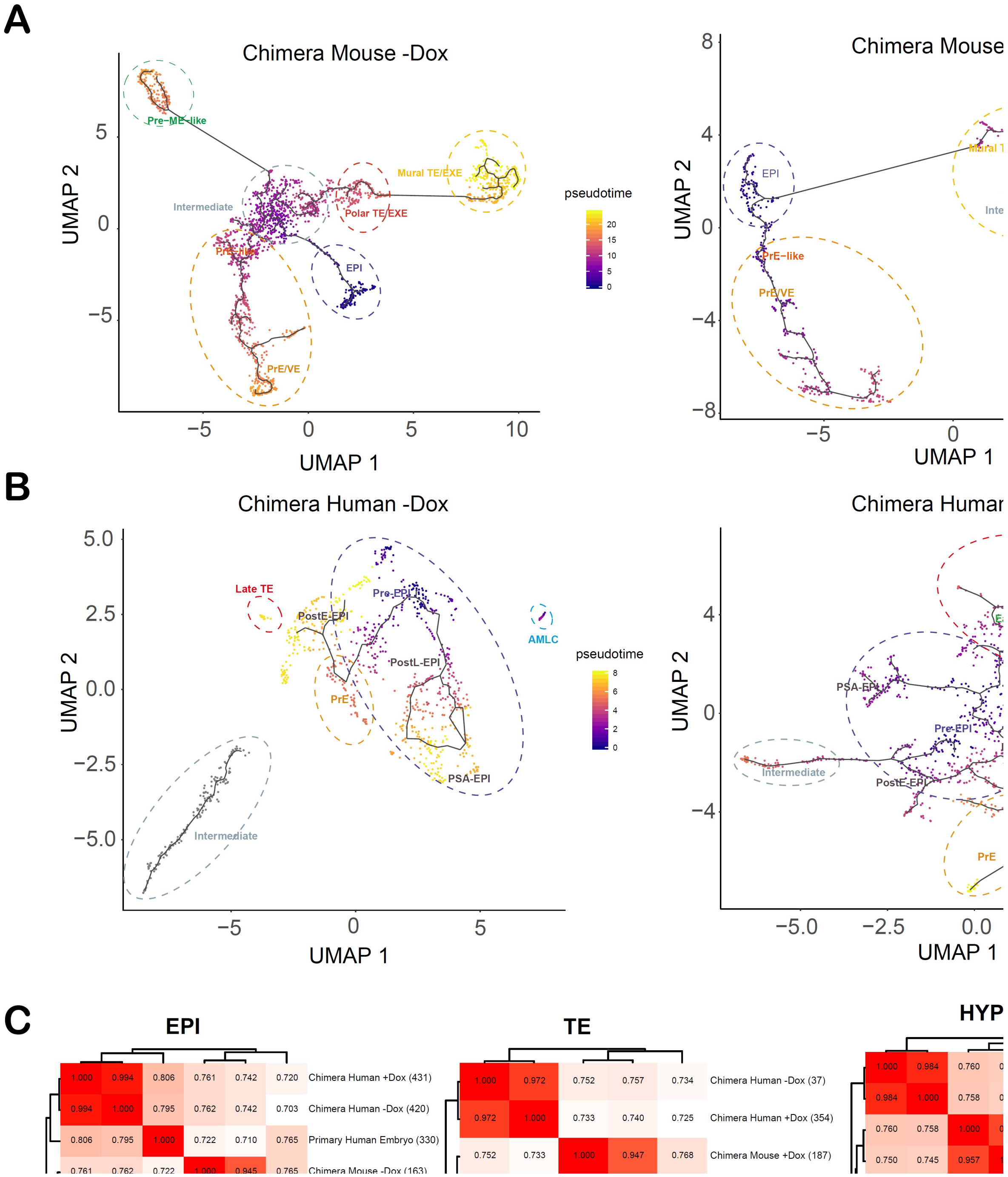

Figure S7. Developmental trajectory and lineage correlation analyses in human-mouse chimeras.

(A) Developmental trajectory of all lineages in -Dox and +Dox conditions of chimeric mouse cells.

(B) Developmental trajectory of all lineages in -Dox and +Dox conditions of chimeric human RUES1-EPSCs.

(C) Heatmaps of Spearman correlation coefficients among different cell origins in -Dox or +Dox conditions, chimeric human RUES1-EPSCs [Chimera Human], chimeric mouse cells [Chimera Mouse], non-chimeric human embryo [Primary Human Embryo], non-chimeric mouse embryo [Primary Mouse Embryo] under corresponding lineages (EPI,

TE, HYPO). Numbers in brackets indicate the number of single cells from each dataset contributing to the analysis.

Table S1. shRNA targeting sequences used in this study.

Target	Clone ID	Target sequence	Application
shRFP	TRCN0000072205	GCTCCGTGAACGGCCACGAGT	gene knockdown
shPODXL#1	TRCN0000117017	CCACCTTTACTGGGTTCTAAA	gene knockdown
shPODXL#2	TRCN0000117019	GTCGTCAAAGAAATCACTATT	gene knockdown
shHMGCR#1	TRCN0000233114	CTATGATTGAGGTCAACATTA	gene knockdown
shHMGCR#2	TRCN0000233115	GGTTCTAAAGGACTAACATAA	gene knockdown

Table S2. DNA primer sequences used in qRT-PCR analyses.

Target	Forward sequence (5' – 3')	Reverse sequence (5' – 3')	Application
OCT4	AGCGAACCAGTATCGAGAAC	TTACAGAACCACACTCGGAC	qRT-PCR
NANOG	TGAACCTCAGCTACAAACAG	TGGTGGTAGGAAGAGTAGAG	qRT-PCR
SOX2	AGCTACAGCATGATGCAGGA	GGTCATGGAGTTGTACTGCA	qRT-PCR
PODXL	AAGGCCAGGGGTTCACAT	AGCCTCGCATCCCTCTAACT	qRT-PCR
HMGCR	GTCATTCCAGCCAAGGTTGT	GGGACCACTTGCTTCCATTA	qRT-PCR
GAPDH	CATCACCATCTTCCAGGAGC	ATGCCAGTGAGCTTCCCGTTC	qRT-PCR
ZSCAN4	ATCCACCTGCCTTAGTCCAC	TCGAAGAACTGTTCCAGCCA	qRT-PCR
ARGFX	GCCAGACCATGTTCCCAGAT	TTTGAATCGCCGGTTCCTGA	qRT-PCR
CPHX1	TCTCAGTTGCTTGCTGGTCTC	GGAAACCACCTGAAGTGCCG	qRT-PCR
DPRX	GAAGATGCCAGGCTCAGAGG	AAGGCTGGGGTTTGGGTATG	qRT-PCR
DUXA	GGGCAAGATCAACCTGGTGT	TGTGTAACTGAGAGGCGCTG	qRT-PCR
DUXB	GGCCAAAGAAATTGGGGTTCC	GAGTCAGATGCTGGGACTGG	qRT-PCR
LETUX	AGACACGGCCATCACTAGG	CCAGGACTTACTGGACGAATGT	qRT-PCR
HERVH	GCAGCCTTTCCTTGGTGTTTAA	GCGTGGTCTGACACCTCTGA	qRT-PCR
HERVK	AGAGGAAGGAATGCCTCTTGCA G	TTACAAAGCAGTATTGCTGCCCG C	qRT-PCR
ITGB6	GCAAGCTGCTGTGTGTAAGGAA	CTTGGGTTACAGCGAAGATCAA	qRT-PCR
SEMA3C	ACCCACTGACTCAATGCAGAGG	CAGCCACTTGATAGATGCCTGC	qRT-PCR
ISL-1	GCAGAGTGACATAGATCAGCCT G	GCCTCAATAGGACTGGCTACCA	qRT-PCR
CDX2	TTCACTACAGTCGCTACATCACC	TTGATTTTCCTCTCCTTTGCTC	qRT-PCR
ELF5	AGTCTGCACTGACATTTTCTCAT C	CAGAAGTCCTAGGGGCAGTC	qRT-PCR
CK7	AGGATGTGGATGCTGCCTAC	CACCACAGATGTGTCGGAGA	qRT-PCR
GATA3	TGCAGGAGCAGTATCATGAAGC CT	GCATCAAACAACTGTGGCCAGT GA	qRT-PCR
TFAP2C	TCTTGGAGGACGAAATGAGATG G	GGGCTTCTTTGATGTAGTTCTGC	qRT-PCR
TEAD4	CAGGTGGTGGAGAAAGTTGAGA	GTGCTTGAGCTTGTGGATGAAG	qRT-PCR
TP63	AGAAACGAAGATCCCCAGATGA	CTGTTGCTGTTGCCTGTACGTT	qRT-PCR
CGA	TGCCCAGAATGCACGCTAC	TTGGACCTTAGTGGAGTGGGA	qRT-PCR

CGB	ACCCTGGCTGTGGAGAAGG	ATGGACTCGAAGCGCACA	qRT-PCR
SDC1	GCTGACCTTCACACTCCCCA	CAAAGGTGAAGTCCTGCTCCC	qRT-PCR
HLA-G	CAGATACCTGGAGAACGGGA	CAGTATGATCTCCGCAGGGT	qRT-PCR
GCM1	CCAAATCCAGCGGGTAATCTT	GGTGAATGGTATGCAGGAGAC	qRT-PCR

Table S3. DNA oligo sequences for targeting PODXL in generating inducible PODXL knockout experiment.

**Target**	**Sequence**	**Status**	**Efficiency**
PODXL sgRNA1	GCTCCGCCCTGGAGCGCGAC	OK	93%
PODXL sgRNA3	GTGTGACCCCGGCGGTGATA	OK	87%
**Target**	**Forward sequence (5' – 3')**	**Reverse sequence (5' – 3')**	**Application**
PODXL-KO(1/2)	AGGCCGTGAATGGTTATCAG	GTGGATGGTGCAAGGTCAG	genotyping
PODXL-KO(7/8)	AGAGGCAGGTTTGCCACTTA	GAGCTGACTCTGGCTGTTCC	genotyping
